# Specific adsorption sites and conditions derived by thermal decomposition of activated carbons and adsorbed carbamazepine

**DOI:** 10.1038/s41598-020-63481-y

**Published:** 2020-04-21

**Authors:** Daniel Dittmann, Paul Eisentraut, Caroline Goedecke, Yosri Wiesner, Martin Jekel, Aki Sebastian Ruhl, Ulrike Braun

**Affiliations:** 1 Bundesanstalt für Materialforschung und -prüfung (BAM), Unter den Eichen 87, Berlin, 12205 Germany; 2 Technische Universität Berlin, Water Quality Control, Straße des 17. Juni 135, Berlin, 10623 Germany; 3 German Environment Agency (UBA), Section II 3.1, Schichauweg 58, Berlin, 12307 Germany

**Keywords:** Chemical engineering, Pollution remediation, Civil engineering, Materials science, Pollution remediation

## Abstract

The adsorption of organic micropollutants onto activated carbon is a favourable solution for the treatment of drinking water and wastewater. However, these adsorption processes are not sufficiently understood to allow for the appropriate prediction of removal processes. In this study, thermogravimetric analysis, alongside evolved gas analysis, is proposed for the characterisation of micropollutants adsorbed on activated carbon. Varying amounts of carbamazepine were adsorbed onto three different activated carbons, which were subsequently dried, and their thermal decomposition mechanisms examined. The discovery of 55 different pyrolysis products allowed differentiations to be made between specific adsorption sites and conditions. However, the same adsorption mechanisms were found for all samples, which were enhanced by inorganic constituents and oxygen containing surface groups. Furthermore, increasing the loadings led to the evolution of more hydrated decomposition products, whilst parts of the carbamazepine molecules were also integrated into the carbon structure. It was also found that the chemical composition, especially the degree of dehydration of the activated carbon, plays an important role in the adsorption of carbamazepine. Hence, it is thought that the adsorption sites may have a higher adsorption energy for specific adsorbates, when the activated carbon can then potentially increase its degree of graphitisation.

## Introduction

Increasing amounts of pharmaceuticals enter the water bodies and are detected all over the world^1–3^. Many of them are persistent and may accumulate within water cycles^4–6^. These organic micropollutants already show adverse effects in aquatic ecosystems despite their comparably low concentrations^7–9^. Therefore, technical measures are introduced as advanced wastewater treatment^10^. One of the favoured techniques is the removal by adsorption onto activated carbon^11,12^.

Activated carbon has been used in water purification for more than hundred years. In modern times, we face more ecological and economical challenges that require the accurate prediction of removal efficiencies prior to the upgrade of wastewater treatment plants. Hence, several approaches have been applied to gain fast and efficient predictions through laboratory experiments and the optimization of operating parameters through data driven modeling^13–16^. The removal of the target substances has been shown to be strongly influenced by the composition of the waters and that of the activated carbons used in the removal^17,18^.

The underlying adsorption processes have been extensively described in theory and investigated experimentally^19,20^. However, adsorption in the liquid phase has been proven to be much more complicated than adsorption in gas phases, especially in complex water matrices that are governed by the presence of dissolved organic matter, the ionic strength and the pH value^21–23^. Hence, both the adsorbate solution and the adsorbent, are mutually influenced. Moreover, activated carbon has a highly heterogeneous pore structure, easily observed by the shape of the adsorption isotherms obtained that are relatively consistent with Freundlich’s model^24,25^. Consequently, there are various adsorption sites that can act as reactive centres with specific adsorption energies/affinities, meaning that this can become an obstacle when trying to investigate different adsorption sites individually. To unravel the underlying mechanisms, researchers have varied solution parameters and modified the carbon surfaces, forming black-box approaches that have not provided sufficient information that is needed for the detailed prediction of adsorption in complex water matrices.

In this study, an approach for the characterisation of micropollutants adsorbed on activated carbons, rather than the analysis of changes in the solution, is proposed. Direct analyses of the inner surfaces of activated carbons are unfeasible, due to the complex adsorption processes and the low concentration of the adsorbates on the adsorbents that predominantly consist of carbon. Therefore, pyrolytic decomposition is applied to the solid sample, upon which the sample’s mass loss is recorded by thermogravimetric analysis (TGA) and the decomposition gases are examined by evolved gas analysis.

As adsorbate model systems, three commercially available powdered activated carbons with differing properties were loaded with varying amounts of carbamazepine. Carbamazepine was selected due to its persistence and abundance in the aquatic environments, in remarkable concentrations, and as it is considered as an anthropogenic wastewater tracer^6,26^. Furthermore, it adsorbs very well onto activated carbon, even under competitive conditions, and which has been shown extensively in literature for adsorption studies^13,17,18,22,27–29^ and also in soil and sediment science^30,31^. Additionally, transformation products of carbamazepine were extensively studied by electrochemical and ozone oxidation, photodegradation and metabolic pathways in water, soil and humans^32–38^. Nielsen *et al*. (2014) conducted thermogravimetry studies coupled with mass spectrometry (TGA-MS) on very similar systems of carbamazepine and two activated carbons. They were able to observe the extensive surface reactions and transformation of carbamazepine during adsorption but could not identify the decomposition products^28^.

To elucidate the underlying mechanisms at work, this study utilises evolved gas analyses through Fourier-transform infrared spectroscopy (TGA-FTIR) to identify the release of small molecules (e.g. H_2_O, CO_2_ or NH_3_), and thermal extraction, combined with thermal desorption gas chromatography and mass spectrometry (TED-GC/MS) to detect larger molecules present in trace concentrations^39^. This can allow for the derivation of thermal decomposition mechanisms and deduce adsorption conditions. Furthermore, specific adsorption sites for the different activated carbons may be accessible by the investigation of thermal decomposition products. This will expand adsorption theories and facilitate the development of models for more accurate predictions.

## Materials and Methods

### Carbamazepine

Carbamazepine (Sigma Aldrich, Germany) was purchased as a certified reference material with a purity of 99.9%. Working solutions of carbamazepine were prepared by dissolving appropriate amounts of the substance in ultra-pure water to reach target concentrations of 1, 5 and 20 mg L^−1^.

### Activated carbons

The powdered activated carbons SAE Super (Norit/Cabot, USA), HK 950 (Carbon Service & Consulting, Germany) and CCP 90D (Donau Carbon, Germany) were used as adsorbates. Table 1 provides detailed material characteristics. Results for the unloaded activated carbons were determined with samples treated like the loaded (see below) but with ultra-pure water instead of carbamazepine working solution.

**Table 1:** Characteristics of the three powdered activated carbons as supplied by the manufacturers^49^, ratios of carbon to hydrogen (C/H ratio), by elemental analyses (unpublished data), ash contents at 900 °C and the molar mass fraction of elements determined by XRF (uncertainty of measurement is negligible for the rounded values).

		SAE Super	HK 950	CCP 90D
Manufacturer		Norit/Cabot	Carbon Service & Consulting	Donau Carbon
Raw material^49^		mixture	charcoal	coconut husk
BET surface area^49^	m^2^ g^−1^	1050–1150	>950	1000–1100
C/H ratio		790	37	300
Ash content		9%	4%	4%
Si	*μ* mol g^−1^	57	160	220
P	*μ* mol g^−1^	1	180	14
S	*μ* mol g^−1^	210	8	7
K	*μ* mol g^−1^	54	19	310
Ca	*μ* mol g^−1^	1900	15	92
Ti	*μ* mol g^−1^	14	3	9
Fe	*μ* mol g^−1^	370	20	79

### X-ray fluorescence spectroscopy

For the characterisation of the inorganic constituents of the activated carbons, the samples were measured using energy-dispersive X-ray fluorescence spectroscopy. The measurements were performed on a Spectro XEPOS III with the software X-LabPro 5.1/TurboQuant (Spectro Analytical Instruments, Germany). The instrument was equipped with 50-W Pd end-window X-ray tube, three polarisation and secondary targets (highly oriented pyrolytic graphite crystal for exciting the elements Al to Cl, K to V, Al_2_O_3_ polarisation target for exciting the elements Zr to Ba and Mo, secondary target for exciting the K-lines of Cr to Y and L-lines of Ta to U) and silicon drift detector. For the measurements, at least 1 g of the powdered activated carbons was placed in an XRF cell (inner diameter of 24 mm) that was covered with 4 *μ*m Mylar film. For semi-quantitative analyses of inorganic constituents in the samples, the fundamental parameters method was applied, which is integrated in the TurboQuant method. For quantitative results (Table 1) the relative amounts were calculated based on the ash content assuming Al as Al_2_O_3_, Si as SiO_2_, P as P_2_O_5_, S as SO_3_, K as K_2_O, Ca as CaO, Ti as TiO_2_ and Fe as Fe_2_O_3_.

### Loading of the activated carbons with carbamazepine

For the adsorption experiments 100 mg of the individual activated carbons were weighed into a 1 L glass bottle and 950 mL of the carbamazepine working solution, with concentrations of 1, 5 or 20 mg L^−1^, were added. 50 mL of the working solutions were kept to determine the initial carbamazepine concentration using UV/vis or LC-MS/MS. The samples were stirred at room temperature for a contact time of 80 min. Afterwards the activated carbons were filtered using cellulose nitrate membrane filters (0.45 *μ*m). 50 mL of the filtrate were used to determine the final carbamazepine concentration in the solution. The loaded activated carbons on the filters were dried in a desiccator overnight. Prior to TGA measurements, the dry samples were detached from the filters.

### Determination of the loadings

To calculate the carbamazepine loading on the activated carbon the initial and remaining concentrations of the analyte in the aqueous solution were determined by UV/vis spectroscopy and liquid chromatography coupled to tandem mass spectrometry (LC-MS/MS). The UV/vis measurements were performed without dilution on a Lambda 12 (PerkinElmer, USA), equipped with a 10 mm Suprasil quartz cuvette (Hellma, Germany) at a wavelength of 284 nm.

Additionally, the LC-MS/MS system TSQ Vantage triple quadrupole mass spectrometer (Thermo Scientific, USA) was used. The chromatographic separation was achieved using a XSelect HSS T3 column (2.1 × 50 mm, 2.5 μm particle size, Waters, USA) with a flow rate of 0.5 mL min^−1^. For carbamazepine the mass to charge ratios (m/z) 194.1 and 179.1 were analysed and quantified via the internal standard consisting of deuterated carbamazepine (D8). Details about the chromatographic method and the applied parameters for the mass spectrometric detection are published elsewhere^13^.

### Microscopic and spectroscopic characterisation

For additional characterisation diffuse reflectance infrared Fourier-transform spectroscopy (DRIFTS) and scanning electron microscopy (SEM) was applied on loaded and unloaded activated carbon samples. The results are provided in the supplementary information and confirm adsorption on the inner surface of the activated carbons without altering the particle morphology.

### Thermogravimetric analyses

**Figure 1:**
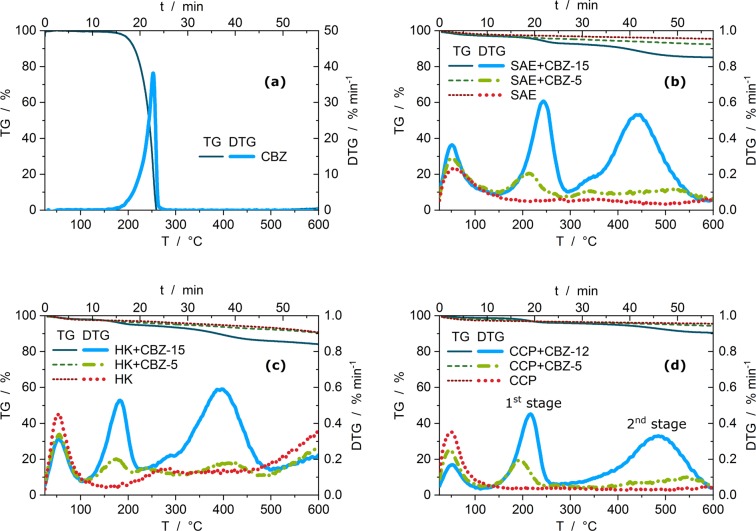
TGA decomposition graphs showing relative sample masses (TG) and mass loss rates (DTG) of (**a**) pure carbamazepine and (**b**) SAE Super, (**c**) HK 950 and (**d**) CCP 90D containing none, middle and high mass fractions of carbamazepine.

**Figure 2:**
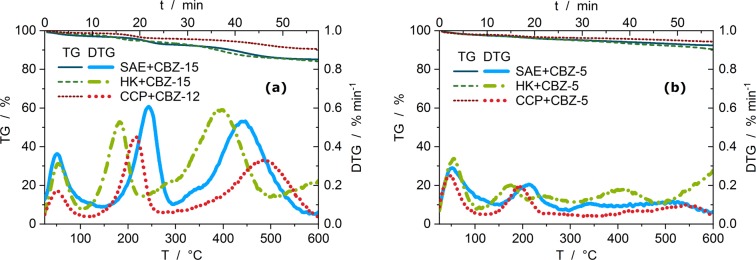
Comparisons of TGA decomposition graphs (from Fig. 1) for the different loaded activated carbons; showing relative sample masses (TG) and mass loss rates (DTG) of (**a**) the systems with the highest carbamazepine loadings and (**b**) the systems with 4.5% mass fraction of carbamazepine.

The thermogravimetric measurements were conducted with the thermobalances TGA/DSC 3+ and TGA 2 (Mettler Toledo, USA) in 150 μL alumina crucibles from 25–600 °C with a heating rate of 10 °C min^−1^. Sample masses of approximately 20 mg for SAE Super and CCP 90D systems were analysed. Due to the low bulk density, only 15 mg of systems with HK 950 were used (this does not alter the DTG profiles and DTG_max_ temperatures, Fig. S1). An inert purge gas flow of 50 mL min^−1^ nitrogen (5.0) ensured pyrolytic conditions. Air buoyancy correction were applied by subtracting an empty crucible measurement or automatic compensation by TGA 2. Plotted DTG data (1 s resolution) in Figs. 1 and 2 are smoothed by Savitzky-Golay with polynomial order of 2 and 59 points of window.

### Determination of decomposition kinetics

Ramp kinetics were conducted with the thermobalance STA-7200 (Hitachi, Japan) in 90 μL alumina crucibles with 5 mg sample mass. A nitrogen purge gas flow of 200 mL min^−1^ is applied for this type of balance. Heating rates of 1, 2, 5 and 10 °C min^−1^ were assessed and evaluated by Friedman’s model free differential isoconversion method using the software NETZSCH Thermokinetics 3.1 (NETZSCH, Germany). Previously, TGA results were subtracted by the mass losses of the corresponding unloaded activated carbon to assess the decomposition stages of carbamazepine solely.

### Evolved gas analyses

Analyses of the evolved decomposition gases during pyrolysis were performed by direct coupling with a Fourier-transform infrared spectrometer or by TED-GC/MS.

### Fourier-transform infrared spectroscopy

TGA-FTIR was achieved by coupling the TGA/DSC 3+ with Nicolet iS50 (Thermo Fisher Scientific, USA) by heated transfer line (250 °C) and IR cell (260 °C). 16 scans per spectrum were collected with 4 cm^−1^ resolution in the range of 4000–400 cm^−1^ using the DTGS detector. Spectra identification and release rates evaluation were performed in the software OMNIC 9.7.39 with the database Nicolet FT-IR Vapor Phase Spectral Library (Thermo Fisher Scientific, USA).

### Thermal extraction-desorption gas chromatography mass spectrometry

TED-GC/MS was conducted with the TGA 2 for the thermal extraction. The decomposition products of the sample were transferred by the nitrogen purge gas flow to a coupling device (Gerstel and BAM, Germany) and trapped on a polydimethylsiloxane solid phase SorbStar (Mercury Instruments, Germany). The use of the solid phase implies selectivity towards the decomposition products, therefore, (polar) substances may pass and will not be detected. The Multi-PurposeSampler MPS (Gerstel, Germany) allows to insert the solid phase at defined temperature ranges during the TGA run. For the measurements presented in this study, the decomposition products were collected between 300 and 600 °C. For sample HK+CBZ-5, an additional sampling step between 25 and 300 °C was performed previously. For the desorption process the loaded solid phase was transported by the autosampler to the thermal desorption gas chromatograph 7890 GC System with mass spectrometer 5977B MSD (Agilent Technologies, USA). The detailed conditions of the GC/MS measurements and further information on the TED-GC/MS can be found elsewhere^39,40^. In this study no internal standard was applied and no quantification was carried out. Peak areas were divided by the sample mass of the respective sample pyrolysed in TGA, only the arising peak areas greater than 10^6^ were taken into account. Due to this selection numbering of the substances is not continuously in the text, but all proposed substances are reported in the supplementary information.

## Results

### Characterisation of the activated carbons

Properties of the investigated powdered activated carbons HK 950, CCP 90D and SAE Super are listed in Table 1. The activated carbons are based on charcoal, coconut husk and a mixture containing lignite, respectively and therefore show large differences. The manufacturers specify similar BET surface areas, but elemental analyses show strong contrasts in the degree of dehydration (cf. C/H ratios). The ash contents also differ, representing the presence of inorganic impurities that are dominated by calcium and iron in the SAE Super. In contrast, phosphorus is dominating in the HK 950, indicating chemical activation with phosphoric acid^41^. Potassium is dominant in the CCP 90D, which is typcial for virgin activated carbons based on coconut husk. Furthermore, the presence of a high sulphur content in the SAE Super is clearly evident and may originate from the lignite portion. Thermal activation can be assumed for the SAE Super and the CCP 90D.

### Characterisation of the thermal decomposition processes

The following sample nomenclature is utilised, where the short names of the activated carbons is used, + carbamazepine (CBZ) if adsorbed with its corresponding mass fraction on a percentage base. Table 2 lists the created adsorbate systems with the thermogravimetric results. Figure 1 shows the decomposition behaviours during TGA of the investigated samples. TGA graphs depict decreasing relative sample mass as thermogravimetry curve (TG) and its mass loss rate (first derivation of TG) as derivative thermogravimetry (DTG) profile as a function of temperature or measurement time, respectively. Figure 1a shows the thermal decomposition of pure carbamazepine within a single stage. The systems with the activated carbons (Fig. 1b–d) decompose differently and show more complex DTG profiles. The two noticeable decomposition stages can be attributed to carbamazepine in the adsorbed state.

**Table 2:** Sample code, mass fractions (w_CBZ_) and molar mass fraction of carbamazepine are given for the created adsorbate systems. Followed by thermogravimetric results with mean values of the mass loss for the 1^st^ and 2^nd^ stage and their share on w_CBZ_. Uncertainties are given as standard deviation, in cases of n = 2 it represents the range of the duplicate determination.

sample	w_CBZ_	CBZ in μmol g^−1^	1^st^ mass loss stage in %	2^nd^ mass loss stage in %	Share of 1^st^ stage on w_CBZ_ in %	Share of 2^nd^ stage on w_CBZ_ in %	n
SAE+CBZ-15	0.150	630	3.8 $\pm$ 0.2	7.1 $\pm$ 0.4	20 $\pm$ 2	38 $\pm$ 3	7
SAE+CBZ-5	0.045	190	1.8 $\pm$ 0.2	2.7 $\pm$ 0.1	21 $\pm$ 4	30 $\pm$ 5	4
SAE+CBZ-1	0.010	42	0.9 $\pm$ 0.0	1.5 $\pm$ 0.0	15 $\pm$ 9	11 $\pm$ 9	3
SAE	0	0	0.8 $\pm$ 0.0	1.4 $\pm$ 0.1	—	—	5
HK+CBZ-15	0.150	630	3.2 $\pm$ 0.2	9.3 $\pm$ 0.1	15 $\pm$ 1	41 $\pm$ 2	2
HK+CBZ-5	0.045	190	1.6 $\pm$ 0.1	4.2 $\pm$ 0.1	16 $\pm$ 4	23 $\pm$ 4	4
HK	0	0	0.9 $\pm$ 0.1	3.1 $\pm$ 0.1	—	—	4
CCP+CBZ-12	0.124	525	2.7 $\pm$ 0.1	5.6 $\pm$ 0.1	16 $\pm$ 1	35 $\pm$ 2	3
CCP+CBZ-5	0.045	190	1.4 $\pm$ 0.1	2.1 $\pm$ 0.1	15 $\pm$ 4	19 $\pm$ 4	4
CCP	0	0	0.7 $\pm$ 0.2	1.3 $\pm$ 0.1	—	—	4

The mass loss up to 130 °C is governed by the vaporisation of adsorbed water. These amounts may change with varying ambient humidity and, therefore, the given values in Table 2, as well as all calculations, are based on values normalised to that of the dry sample masses after the vaporisation stage. Pyrolysis of the unloaded activated carbons (red dotted lines in Fig. 1b–d) shows specific DTG profiles. These may be interpreted as baselines for each activated carbon and represent functional groups and oxygen containing surface complexes released from the adsorbent^42^.

Two additional mass loss stages occur during pyrolysis of the loaded activated carbons compared to that of the unloaded adsorbents. The temperatures at which carbamazepine decomposes are observed. The 1^st^ decomposition stage of carbamazepine, adsorbed on HK 950 and CCP 90D, begin at lower temperatures (130 °C and 160 °C, respectively) than that of the decomposition of pure carbamazepine (180 °C) or in the SAE Super systems. The 2^nd^ decomposition stage takes place between 300 and 600 °C. At these temperatures pure carbamazepine is already decomposed completely.

In Fig. 2a (and Table S1) the thermal decomposition of the systems with the highest carbamazepine loadings are compared. No systematic shift in the decomposition temperature is observed to be dependent upon the activated carbon. The 1^st^ and the 2^nd^ mass loss stages shift independently to higher or lower temperatures.

There is no complete pyrolytic release of adsorbed carbamazepine. For SAE+CBZ-1, 74% of the initial carbamazepine mass is retained and represents 0.74% of mass fraction in the sample. For SAE+CBZ-15, only 42% of the initial carbamazepine mass is retained but this represents 6.3% of mass fraction in the sample. These are remarkable amounts considering the complete decomposition of pure carbamazepine.

Furthermore, the 2^nd^ stage’s mass loss of adsorbed carbamazepine decreases disproportionally with carbamazepine mass fraction and may disappear at very low loadings. Therefore, the increasing retention of carbamazepine at low loadings indicates high adsorption energies for the favoured adsorption sites. On the other hand, the increasing mass fraction of retained carbamazepine at high loadings indicates inter-molecular interactions, e.g. pore filling. In conclusion, high-energy adsorption sites dominate the incomplete release of carbamazepine at low loadings while pore filling may cause the high retention at high loadings.

### Decomposition mechanism of the 1^st^ mass loss stage

**Figure 3:**
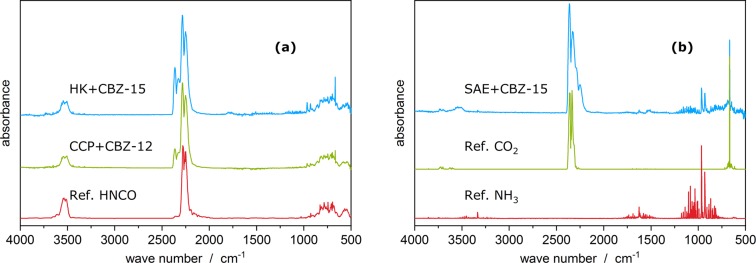
Infrared spectra of the gases evolving during the 1^st^ decomposition stage. From top to bottom (blue – green – red): (**a**) spectra at 16.1 min and at 19.5 min as well as reference spectrum of isocyanic acid (HNCO); (**b**) spectrum at 22.3 min and reference spectra of carbon dioxide (CO_2_) and ammonia (NH_3_). The water spectrum was subtracted from the measured spectra.

The main decomposition product evolved during the 1^st^ decomposition stages is isocyanic acid, which was identified by TGA-FTIR (Fig. 3). Isocyanic acid is a likely leaving group of carbamazepine. Furthermore, the elimination of isocyanic acid during the thermal decomposition of pure carbamazepine has been previously described by Pinto *et al*.^43^. CO_2_ and NH_3_ are released simultaneously as isocyanic acid may be hydrolysed. This secondary reaction is intensified on SAE Super and might be catalysed by its comparably high content of 9% inorganic compounds (containing Ca, Ti and Fe; see Table 1). A detailed description of this mechanism, and evidence for H_2_O consumption, is given in the supplementary information (Figs. S2 and S3).

We conducted experiments on decomposition kinetics for SAE+CBZ-15 and HK+CBZ-15, to investigate catalytic effects that may cause the observed temperature shift for the elimination of isocyanic acid in the 1^st^ decomposition stages (Fig. 2 and Table S1). As a result, we determined activation energies of 92 $\pm$ 2 kJ mol^−1^ for SAE+CBZ-15 and 92 $\pm$ 3 kJ mol^−1^ for HK+CBZ-15 very close to 93 $\pm$ 2 kJ mol^−1^ for pure carbamazepine (Table S2)^43^. In conclusion, no catalytic effects seem to be at play. There is no interaction between the amide group of carbamazepine and the activated carbon surface, as isocyanic acid is eliminated in the different systems without observed changes in the activation energy, in comparison to pure carbamazepine.

**Figure 4:**
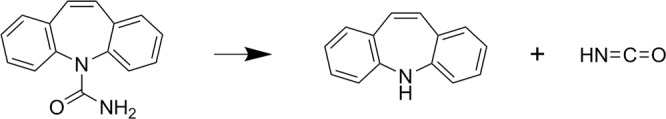
Elimination of isocyanic acid from the carbamazepine molecule with remaining dibenzazepine (60, numbering see supplementary information). This reaction occurs during the 1^st^ decomposition stage of adsorbed carbamazepine and by thermal decomposition of pure carbamazepine. In the latter, both products evolve as gases. For adsorbed carbamazepine, only isocyanic acid is released while dibenzazepine remains on the activated carbon in the 1_st_ stage independent of its loadings or type of adsorbent.

We assume, that the availability of hydrogen atoms in the activated carbons may affect eliminating isocyanic acid at lower temperatures. This hypothesis is supported by the fact that one hydrogen atom has to move from the amide group to the nitrogen atom of the dibenzazepine moiety (Fig. 4). Furthermore, the order of the peak temperature in the DTG profiles of the 1^st^ decomposition stages (Fig. 2 and Table S1) are in good agreement with the order of the C/H ratios of the activated carbons (HK – CCP – SAE, Table 1).

The known mass fraction of carbamazepine in the loaded activated carbons (w_CBZ_) and the mass loss stages gained by TGA are provided in Table 2. The mass share of isocyanic acid in the carbamazepine molecule of 18.2% corresponds to the respective mass loss in the 1^st^ decomposition stage, which are about 16 and 19% of w_CBZ_.

In conclusion, the 1^st^ decomposition stage of adsorbed carbamazepine on activated carbon arises out of the quantitative elimination of isocyanic acid and is observed to be independent of the carbamazepine loading (Fig. 4).

### Decomposition mechanisms of the 2^nd^ mass loss stage

After the elimination of isocyanic acid in the 1^st^ decomposition stage, dibenzazepine molecules should remain adsorbed on the activated carbon. The broad DTG peak of the 2^nd^ mass loss stage (Figs. 1 and 2) indicates a number of overlapping decomposition processes. Nielsen *et al*. (2014) conducted TGA-MS and assumed decomposition products in the 2^nd^ mass loss stage with m/z higher than 100^28^. We could confirm this from evolved gas analyses by TED-GC/MS, revealing 55 products (an overview with all proposed structures of decomposition products is presented in the supplementary information; initially 65 substances were observed, due to several reasons 10 were excluded). The evolved products may not completely originate from the carbamazepine molecule, as 6 substances contained oxygen, 5 sulphur and 27 contained no heteroatoms. However, these substances evolve solely or in increased amounts in systems with adsorbed carbamazepine. In contrast, pure carbamazepine releases mainly dibenzazepine (substance 60 in the SI, only numbers in brackets are given below) as detected by TED-GC/MS and Py-GC/MS^44^. Traces of acridine (54) and 10,11,-dihydro-5H-dibenzazepine (59) are observed, which are in in the first approximation related to a disproportionation reaction of (60). Pyrolysis and the evolution of decomposition products from the loaded activated carbons are driven by the chemical composition of the sample. For the heterogeneous activated carbon, this may be linked to reactive centres with varying adsorption energies for carbamazepine. These centres are called adsorption sites.

**Figure 5:**
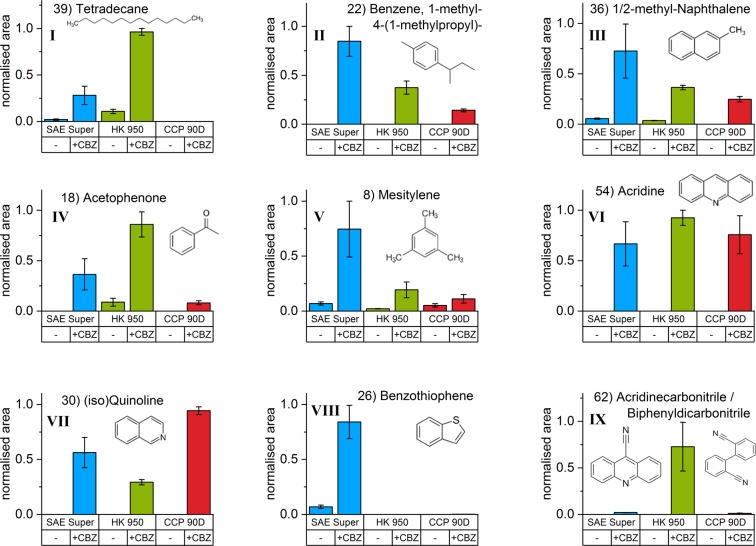
Relative comparison of selected evolved decomposition products of the unloaded and loaded activated carbons with 4.5% mass fraction of carbamazepine. Substances are representing the nine groups (**I–IX**) also shown in Fig. 7. Values are normalised to sample mass and the maximum peak area of the single measurements. Error bars representing the range of the duplicate determination.

We assigned the decomposition products to nine groups (I-IX), based on their general molecular structure and release pattern in the investigated systems. Figure 5 shows selected proposed substances as representatives for each group and provides their relative releases (normalised to sample mass) from the unloaded activated carbons and the systems with 4.5% mass fraction of carbamazepine. Graphs of all 55 substances are provided in the supplementary information.

**Figure 6:**
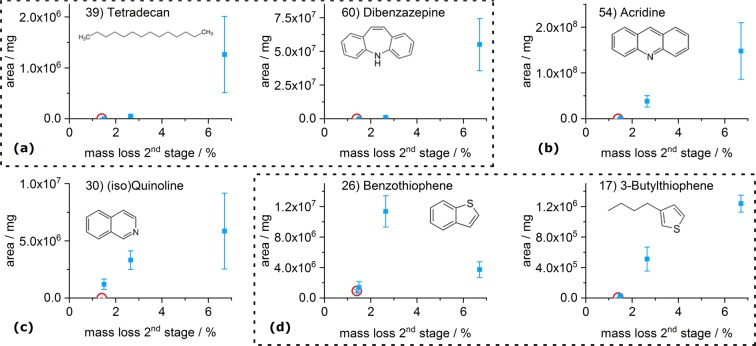
Selected evolved decomposition products of the unloaded SAE Super (red circle) and its systems with varying amounts of adsorbed carbamazepine, represented by the 2^nd^ stage’s mass loss. Types of release curves are shown for (**a**) starting at higher loadings, (**b**) continuously increasing, (**c**) limited at high loadings and (**d**) connected releases, due to hydration at high loadings. Values are normalised to sample mass. Error bars representing the range of the duplicate determination.

I. Tetradecane (39) and the group of *aliphatics* (decomposition products without aromatic moieties) are released predominantly from the HK+CBZ-5 system. They are not detected in the systems with CCP 90D. Figure 6a shows an increasing release of (39) with increasing carbamazepine mass fraction in SAE Super but the release of *aliphatics* is not present at low carbamazepine loadings.
II. Equally, the group of *strongly alkylated benzenes* (decomposition products consisting of a phenyl ring and a substitution with at least four carbon atoms) is progressively released with increased carbamazepine loading. Also, the lowest release occurs from CCP+CBZ-5. Like 1-methyl-4-(1-methylpropyl)-benzene (22), this group is not released from unloaded activated carbons but from all systems with adsorbed carbamazepine.
III. Methylnaphthalene (36) belongs to the group of *alkylated or bridged aromatics* (decomposition products consisting of more than one phenyl ring with substitutions) which is similar to the *strongly alkylated benzene* (II) group with regard to the release order of the three activated carbons.
IV. Substances in the group of *oxygen containing aromatics* (decomposition products consisting of aromatics with substitute oxygen), e.g. acetophenone (18), were released predominantly by HK+CBZ-5. This is in line with the comparably high amounts of oxygen containing surface complexes of HK 950, represented by its pyrolytic mass loss. Furthermore, benzaldehyde (9) was released even at low carbamazepine loadings on SAE Super (see SI) indicating specific surface reactions or adsorption sites.
V. *Slightly alkylated benzenes* (decomposition products consisting of phenyl ring and substitution having at most three carbon atoms) like Mesitylene (8) are found within all investigated systems.
VI. Acridine (54) gained, by far, the strongest intensities. Therefore, it is assumed to be a main decomposition product in the 2^nd^ stage. It is assigned to the group of *unsubstituted aromatics* (aromatic decomposition products without substitutions) as well as dibenzazepine (60). All substances of this group are released in similar amounts from all three activated carbons. Figure 6b shows an increasing release of (54) with increasing carbamazepine mass fraction.
VII. Quinoline (30) is released from CCP+CBZ-5 in the largest quantities and represents the group of *unassigned, nitrogen containing* substances (decomposition products with differing release patterns, mostly containing nitrogen). This group is not released from unloaded activated carbons. Benzonitrile (11) and (30) are released even at low carbamazepine mass fractions in SAE Super (Figs. 6c and SI).
VIII. Benzothiophene (26) is one of five *sulphur containing* decomposition products primarily released from the SAE Super systems, as this activated carbon contains the highest amount of sulphur (Table 1). Furthermore, Benzothiophene (26) shows a maximum with decreasing release after 4.5% mass fraction of carbamazepine whereas 3-butylthiophene (17) is observed to constantly increase (cf. Fig. 6d). In total, the release of *sulphur containing* decomposition products reached a limitation at 4.5% mass fraction of carbamazepine. With regard to the available sulphur of 210 *μ*mol g^−1^ in the SAE Super, it is close to the molar mass fraction of carbamazepine in SAE+CBZ-5 (cf. Tables 1 and 2).
IX. 9-Acridinecarbonitrile (62) is an outstanding and *specific* decomposition product containing two nitrogen atoms. It is released from all systems containing carbamazepine but predominantly by HK+CBZ-5. Additionally, TGA-FTIR revealed the release of hydrogen cyanide (Fig. S4) solely from HK 950 that may facilitate the reaction of acridine (54) to (62). On the other hand at 180 °C water is also released from this system (Fig. S3), which can transform carbamazepine to (62). Subsequently, (62) could release hydrogen cyanide during its thermal vaporisation.

**Figure 7:**
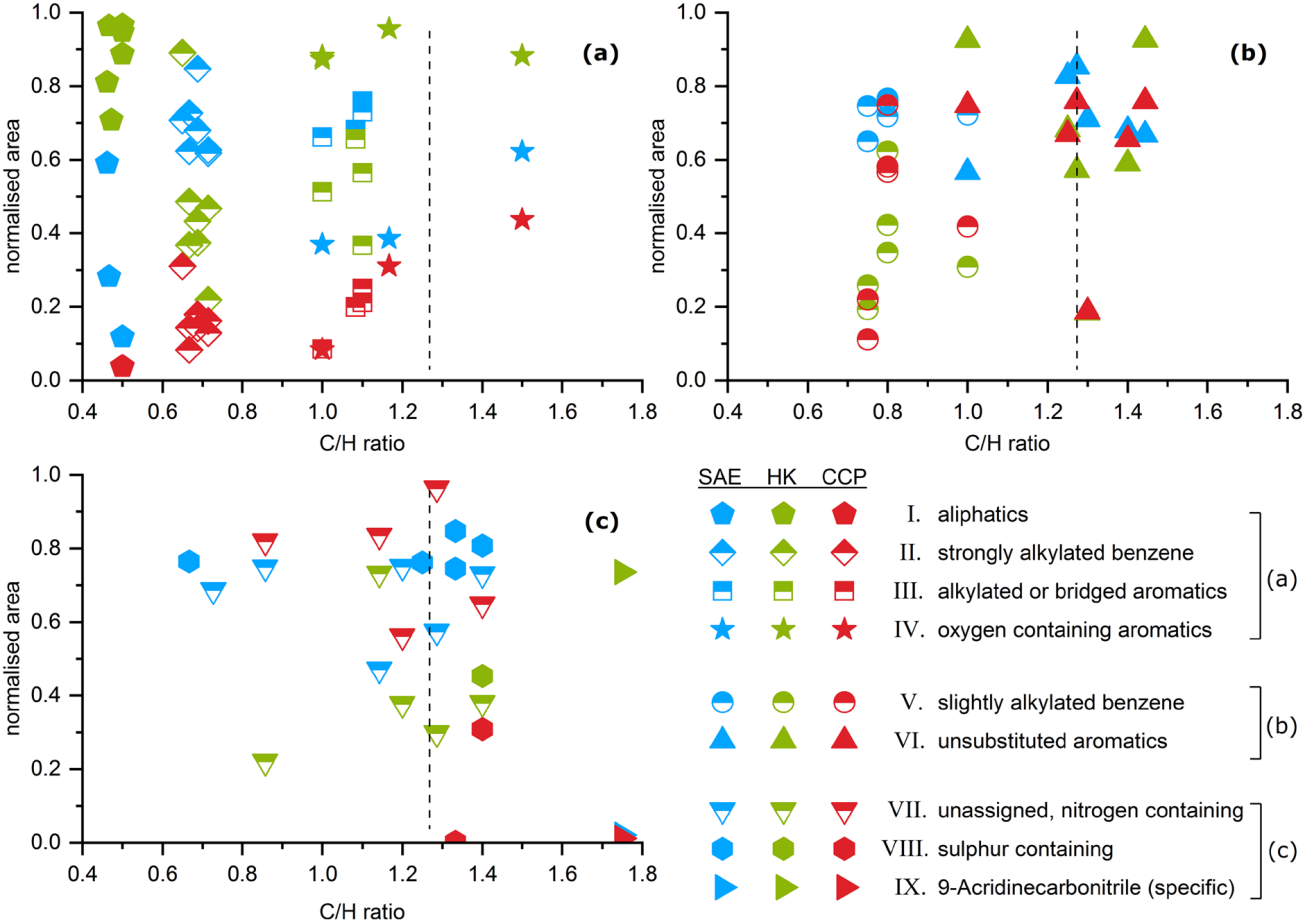
All decomposition products of the systems with 4.5% mass fraction of carbamazepine are plotted by their C/H ratios and normalised peak area, coloured for each activated carbon. Symbols represent the nine groups that are mapped in (**a**) good clustering by activated carbon, in (**b**) less distinctiveness and in (**c**) unassigned and specific released decomposition products. The dashed line indicates the C/H ratio of dibenzazepine (60).

Figure 7 shows the normalized peak areas and C/H ratios of all individual substances arranged in the nine groups. The dashed lines indicate the C/H ratio of dibenzazepine (60) to support differentiation whether the decomposition products become more or less hydrated during pyrolysis.

The *aliphatics* (group I) have low C/H ratios or comparably high hydrogen contents, respectively (Fig. 7a). Therefore, their release depends on receiving hydrogen atoms. The activated carbons have a much higher C/H ratio (Table 1) and are therefore considered as poor H sources. Thus, only carbamazepine can provide the hydrogen required for the observed evolution of hydrogen-rich substances. We attribute the release of *aliphatics* to pore filling and multi-layer formation since their low C/H ratios can only be obtained by inter-molecular reactions. Additionally, their releases evolve at high loadings and therefore decomposition products are not related to chemical properties of the activated carbons like hetero atoms or oxygen containing surface complexes. The group is clearly clustering by the type of activated carbon, showing a prevailing release of HK 950 after carbamazepine adsorption. This might be related to the pore structures supporting, or not supporting, pore filling and multi-layer formation. Also, the evolution of *strongly alkylated benzenes* (group II) depends on the availability of hydrogen provided by the adsorbed carbamazepine. However, clusters of the substances with low C/H ratio are observed in a different order to that seen for *aliphatics*, with respect to the activated carbon type. For *alkylated or bridged aromatics* (group III) and *oxygen containing aromatics* (group IV), a clear differentiation of the activated carbons can be observed. These substances are much closer to the C/H ratio of dibenzazepine (60) and do not require the additional hydrogen atoms provided through inter-molecular reaction. Distinct adsorption sites may be associated to the evolution of *oxygen containing aromatics*. Interactions with the activated carbon surface, are also indicated, as oxygen is not present in the carbamazepine molecule.

Figure 7b shows the groups with non-distinctive clustering of the decomposition products such as *slightly alkylated benzenes* (group V) and *unsubstituted aromatics* (group VI). As they are found in all investigated systems, they can be said to originate from adsorption sites and conditions present in each activated carbon. These substances also indicate specific interactions with the activated carbon surface as their release curves do not increase linearly with increasing carbamazepine loadings, as it is shown for quinoline (30, group VII) in Fig. 6c (cf. SI). There are two exceptions in group VI, acridine (54) shows a linear release curve (Fig. 6b) and dibenzazepine (60) is only released at higher loadings. The intense release of acridine (54) already starts at low carbamazepine loadings and shows no limitation due to the availability of adsorption sites, which may be the most abundant and typical for activated carbons. In contrast, all other substances that are released at low loadings (9, 11, 25, 30, 53) show limitations or a maximum release at 4.5% mass fraction of carbamazepine. We suggest that this represents favoured adsorption sites, and these substances are assigned to the groups IV, VI and VII. In the case of no further increases in release beyond 4.5% mass fraction of carbamazepine, the adsorption conditions related to these decomposition products seem to be limited on the activated carbon surface.

*Unassigned, nitrogen containing* decomposition products (group VII) with a wide variety in C/H ratios are shown in Fig. 7c. The graph also contains all *specifically* released substances (13, group VII and 62, group IX) including *sulphur containing* ones (group VIII), the latter prevail in the SAE Super systems. As shown in Fig. 6d, there is a decrease in the evolution of benzothiophene (26) but an increase of 3-butylthiophene (17). The latter indicates the presence of pronounced hydration reactions with increasing carbamazepine loadings since it can be derived from (26) by hydration. These effects seem to be related to low C/H ratios of the evolved substances. It indicates increasing inter-molecular reactions at high carbamazepine loadings. This has also been observed for the *aliphatics* (group I), where the release only begins at 4.5% mass fraction of carbamazepine. These increasing release curves are similar to others from decomposition products occurring at high carbamazepine loadings, e.g. methyl-acridines (56, 58, 61) or iminodibenzyl (59). Furthermore, dibenzazepine (60) is only released at high loadings but is not hydrated. This may allow the differentiation between hydrated products generated by pore filling and (60), which may be evolved from multi-layers as it is released from pure carbamazepine.

**Figure 8:**
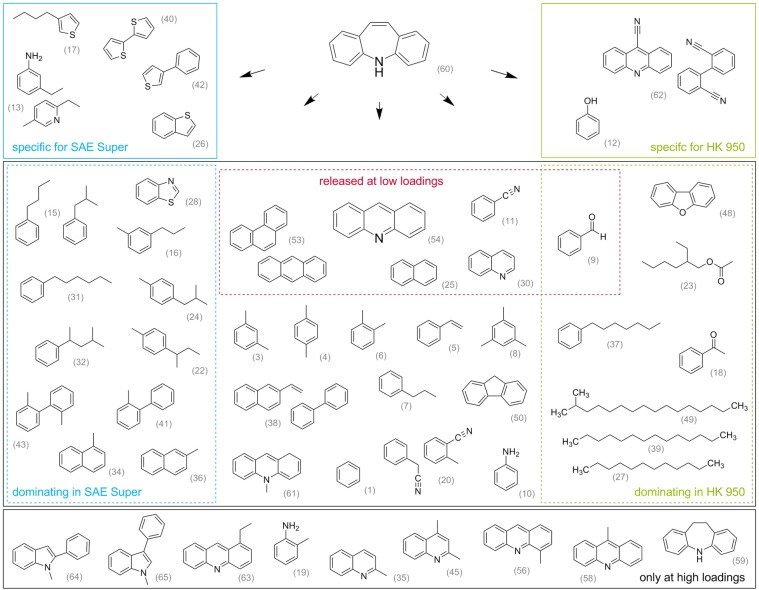
Proposed structures for substances released during the 2^nd^ decomposition stage. Starting with dibenzazepine (60) (“iminostilbene”) adsorbed on activated carbon strongly differing products are formed. Boxes support categorisation of substances which are *specific* for the activated carbons SAE Super or HK 950 as well as substances *dominating* in clustering groups (Fig. 7a). The red dashed box emphasizes decomposition products which are released at low carbamazepine loadings. The gray box on the bottom indicates substances that were released only at high loadings investigated on the SAE Super.

In Fig. 8, all observed decomposition products from the 2^nd^ decomposition stage are arranged according to the examined features. There are specific decomposition products for SAE Super and HK 950 but none for CCP 90D, which has less distinctive properties. Furthermore, systems of CCP 90D do not release products dominating their substance group. The substances solely observed at high loadings from SAE+CBZ-15 are considered to originate from growing pore filling and multi-layer formation. However, the substances released, even at low loadings, are emphasised and are mainly evolved from all adsorbate systems. Therefore, they represent favoured adsorption sites and the generally high affinity of carbamazepine to activated carbons.

## Discussion

The three powdered activated carbons, studied with and without adsorbed carbamazepine, showed a variety of differences in thermal decomposition. However, it was observed that adsorbed carbamazepine decomposes in two stages on all activated carbon samples, within this study, showing that the adsorption mechanisms for carbamazepine is the same for the different activated carbons. In contrast, application of other adsorbents, such as mesoporous silicates with comparable amounts of adsorbed carbamazepine have been reported to decompose with only one stage. Furthermore, complete pyrolytic removal was achieved on these materials, indicating the presence of fewer complex mechanisms occurring in comparison to that occurring with activated carbon^45^.

The quantitative release of isocyanic acid (Fig. 4) in the 1^st^ decomposition stage with all loaded activated carbons supports, indicating comparable adsorption mechanisms for all three adsorbents. Hence, the adsorption of carbamazepine on activated carbon is said to take place mainly at its dibenzazepine moiety. The temperature shift of the 1^st^ decomposition stage can be explained by the differing availabilities of hydrogen atoms in the activated carbons (or hydrogen deficiencies), represented by the C/H ratio. The hydrogen content may be more important for thermal decomposition than for adsorption of carbamazepine in water.

To understand the adsorption process of carbamazepine, the 2^nd^ decomposition stage reveals more information. It provides information on the specific properties of activated carbon, as well as the amount of adsorbed carbamazepine. 55 decomposition products were observed, by TED-GC/MS, during the pyrolytic decomposition of carbamazepine on activated carbon (Fig. 8).

Highly hydrated substances, in the evolved gases, with comparably lower C/H ratios are *dominating* specific activated carbons but are *not released at low carbamazepine loadings*. Therefore, they are said to depend on the activated carbon properties such as pore structure or their degree of dehydration and may represent pore-filling effects. In addition, dibenzazepine (60) and other decomposition products, that were observed *only at high loadings*, may represent multi-layer formation.

Sulphur or oxygen containing decomposition products indicate interactions of carbamazepine with heteroatoms or functional groups on the activated carbon surface, or with inorganic constituents. The latter may be Fe_x_S_y_ that exchange to iron nitrates or cyanides and therefore, favour the presence of carbamazepine’s nitrogen. The evolved substances are strongly associated with the activated carbon properties such as the amount of oxygen containing surface complexes or inorganic impurities, and hence, they may provide additional adsorption sites (cf. HK 950 and SAE Super).

The majority of decomposition products evolve from all three activated carbons despite the strongly differing adsorbent properties. This is in line with the similar TGA decomposition processes observed and the fact that carbamazepine is very well adsorbed on all activated carbons. Moreover, the substances *released at low loadings* (9,11,25,30,53,54) were observed with all three activated carbons. In conclusion, the favoured adsorption sites for carbamazepine are observed in all three activated carbons but present in differing amounts. Acridine (54) was the decomposition product with the most intense response during pyrolysis. This is also a well known transformation product of carbamazepine from electrochemical oxidation, photodegradation and metabolic pathways^32,34–37^. There are no comparable transformation products by ozonation^33^.

Desorption studies in water indicate the release of intact carbamazepine molecules as well as desorption hystereses from activated carbons, graphene oxides and soils^30,31,46,47^. For thermal desorption or decomposition, mass balances showed that dibenzazepine is partialy retained on the activated carbon support. Therefore, the decomposition products stem only from a fraction of the adsorbed carbamazepine. Moreover, there may be a high number of substances associated with the carbon structure of the activated carbon, rather than that of the carbamazepine molecule.

As a result, we can propose a hypothesis on these phenomena. During the formation of activated carbons as technical materials, hydrocarbons are dehydrated at 800–1000 °C and highly condensed aromatic systems arise (cf. C/H ratios). Subsequently, activated carbons are non-graphitic and non-graphitisable, meaning they will not reach the thermodynamically preferred state of graphite, even upon heating to 3000 °C^25^. However, activated carbon’s highly disordered microstructure contains small graphite-like crystallites. Due to steric hindrance they are not able to become graphite within the carbon skeleton and there are no possible pathways for ongoing dehydration processes to proceed. An adsorbate could act as supporting structure donating hydrogen and could thus facilitate dehydration. In conclusion, adsorption sites of high adsorption energy, for carbamazepine, would represent regions in the activated carbon structure that have a strong dependence or high possibility to become more graphite-like. On the other hand, continual retention of carbamazepine mass fraction with increasing loadings are attributed to pore-filling effects.

In brief, adsorbates could support the closing of voids between graphite-like crystallites or reduce defects by squeezing out heteroatoms from the activated carbon. Therefore, strongly adsorbing molecules like carbamazepine are partially integrated in the activated carbon structure.

Future work is needed to verify the proposed hypothesis, keeping in mind the chemical composition of an activated carbon, especially the C/H ratio, in addition to pore structure and surface area. This will be a challenge for further investigations with more complex water matrices containing micropollutants. Other than this, the proposed approach could help in improving carbonaceous adsorbent materials, whilst providing further understanding in regeneration processes.

## Supplementary information

Supplementary Information.

Supplementary Information2.

## Data Availability

The datasets generated and analysed during the current study are available in the zenodo repository^48^.
