# Supplementary material for: Specific adsorption sites and conditions derived by thermal decomposition of activated carbons and adsorbed carbamazepine
Source: Sci Rep. 2020 Apr 21;10:6695. doi: 10.1038/s41598-020-63481-y (PMC7174341; doi:10.1038/s41598-020-63481-y)
Supplement: Supplementary file 1 — Supplementary Information. [file 41598_2020_63481_MOESM1_ESM.pdf]

Supplementary information for "Specific adsorption sites and conditions derived by thermal decomposition of activated carbons and adsorbed carbamazepine"

Daniel Dittmann[1,2,*], Paul Eisentraut[1], Caroline Goedecke[1], Yosri Wiesner[1], Martin Jekel[2], Aki Sebastian Ruhl[2,3], and Ulrike Braun[1]

[1]Bundesanstalt für Materialforschung und -prüfung (BAM), Unter den Eichen 87, Berlin, 12205, Germany
[2]Technische Universität Berlin, Water Quality Control, Straße des 17. Juni 135, Berlin, 10623, Germany
[3]Umweltbundesamt, Schichauweg 58, Berlin, 12307, Germany
[*]daniel.dittmann@tu-berlin.de

ABSTRACT

The supplementary information consists of this PDF file and a spreadsheet named *Structures_by_TED-GC-MS* (provided in XLSX and ODS) containing all information about the decomposition products detected by TED-GC/MS. Additionally, the whole raw data is provided within the zenodo repository.[1] This PDF file contains

1. TGA decomposition stages
2. Hydrolysis of isocyanic acid
3. Decomposition products of the 2nd mass loss stage
4. SEM comparisons
5. DRIFTS comparisons
References
Acknowledgements

1 TGA decomposition stages

The TGA graphs shown in Figures 1&2a are characterised by two decomposition stages after carbamazepine adsorption. Table S1 provides the temperature ranges and the temperatures of maximum mass loss rate (DTG_{max}) for each mass loss stage. The temperature ranges were used to determine the mass losses in Table 2 and to calculate the kinetic parameters shown in Table 3. DTG_{max} temperatures show the shifts of the decomposition stages depending on the activated carbon as graphically assessed in Figure 2a.

	1st mass loss stage		2nd mass loss stage	
sample	range in °C	DTG_{max} in °C	range in °C	DTG_{max} in °C
SAE+CBZ-15	150-295	242	295-575	446
HK+CBZ-15	103-222	181	222-502	397
CCP+CBZ-12	120-263	216	263-600	487

Table S1. TGA characteristics for the 1st & 2nd mass loss stage of the systems with the highest carbamazepine loadings.

1.1 Influence of sample mass on DTG profiles

The activated carbon HK 950 has a comparable low density and therefore TGA sample mass was 15 mg in the crucibles. This could alter thermal decomposition of samples with 20 mg and parameters like DTG_{max} temperatures in Table S1.

Figure S1 shows the SAE+CBZ-15 system with different sample masses. There is no shift in DTG_{max} temperature of the sample analysed with 15 mg. Therefore, results in Table S1 are not sensitive to the analysed sample masses with slight mass changes.

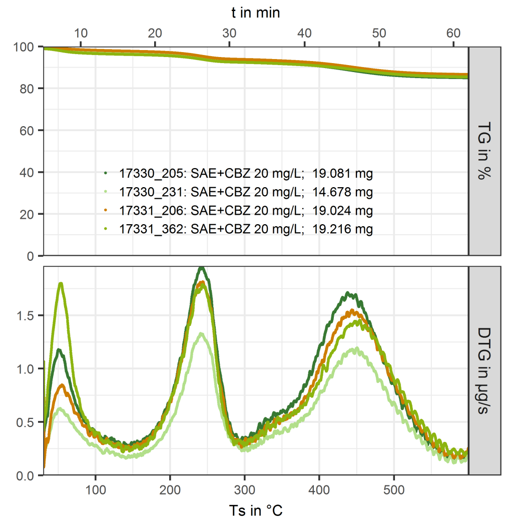

Figure S1. TGA results for the SAE+CBZ-15 system with varying sample masses. No shift in DTG_{max} temperatures can be observed.

1.2 Comparison of kinetic parameters

The kinetic parameters determined by TGA ramp-kinetics are provided in Table S2. The results of Pinto et al. (2014)[2] were conducted by heating rates of 2.5, 5, 10 and 15 °C min^{-1}. We assume that the magnitude of the pre-exponential factor (k) differs, due to experimental conditions including carbamazepine investigated as adsorbate or pure powder.

	Activation energy (E_a)	Pre-exponential factor (k)
SAE+CBZ-15	92±2 kJ mol^{-1}	450 min^{-1}
HK+CBZ-15	92±3 kJ mol^{-1}	540 min^{-1}
Carbamazepine[2]	93±2 kJ mol^{-1}	8.6±0.1 min^{-1}

Table S2. Kinetic parameters determined by ramp kinetic experiments for splitting-off isocyanic acid from carbamazepine.

2 Hydrolysis of isocyanic acid

Isocyanic acid (HNCO) can be hydrolysed to carbon dioxide and ammonia (Figure S2). This reaction was already described at 80 °C and only with traces of water vapour.[3]

$$HNCO + H_2O \longrightarrow CO_2 + NH_3$$

Figure S2. Hydrolysis of isocyanic acid to carbon dioxide and ammonia.

Since we worked with nitrogen as purge gas with 5.0 purity we did not expect noticeable amounts of water. However, we observed the consumption of water in the release rates of TGA-FTIR during HNCO release in SAE+CBZ-15 (yellow line in Figure S3). Simultaneously, the evolution of carbon dioxide and ammonia increased while isocyanic acid decreased.

This was solely found in the TGA-FTIR measurements of the systems with SAE Super, indicating specific catalytic effects by this activated carbon probably by its inorganic inclusions. The amount of water consumed corresponds with the hydrolysis reaction for isocyanic acid. It equals about 0.01% water in the nitrogen purge gas.

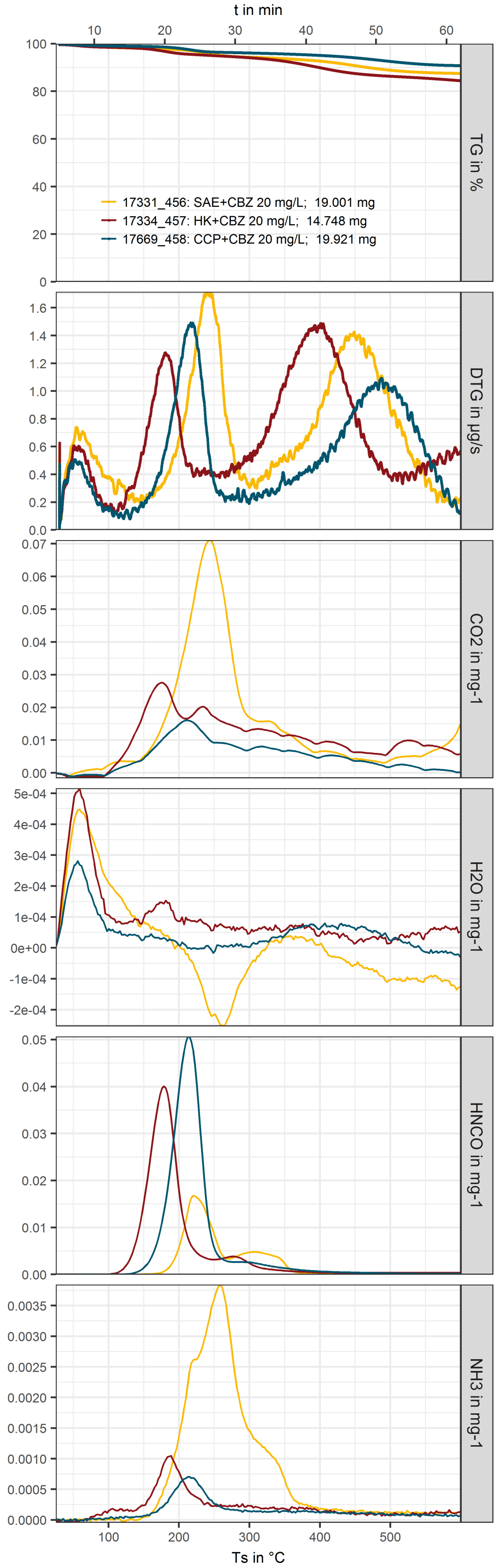

Figure S3. TGA-FTIR results for the highest loadings of carbamazepine on the three activated carbons. The figure corresponds to Figure 2(a). Release rates of carbon dioxide, water, isocyanic acid and ammonia are shown normalised to sample mass.

3 Decomposition products of the 2nd mass loss stage

The decomposition products observed by TED-GC/MS are provided in the spreadsheet *Structures_by_TED-GC-MS* in the supplementary information files. Figures for comparing the systems with 4.5% carbamazepine mass fraction (like Figure 5) and the systems with SAE Super and varying carbamazepine mass fractions (like Figure 6) are additionally shown in section 3.2. First, section 3.1 shows TGA-FTIR results for the release of hydrogen cyanide.

3.1 Release of hydrogen cyanide

A specific characteristic of the systems with HK 950 is the release of significant amounts of hydrogen cyanide (HCN) after carbamazepine adsorption. This was observed by TGA-FTIR and can help to interpret the release of decomposition products observed by TED-GC/MS. Figure S4 shows the TGA-FTIR measurements with HCN release rates of the systems with high carbamazepine loadings. Unloaded activated carbons do not release any HCN at the investigated temperatures.

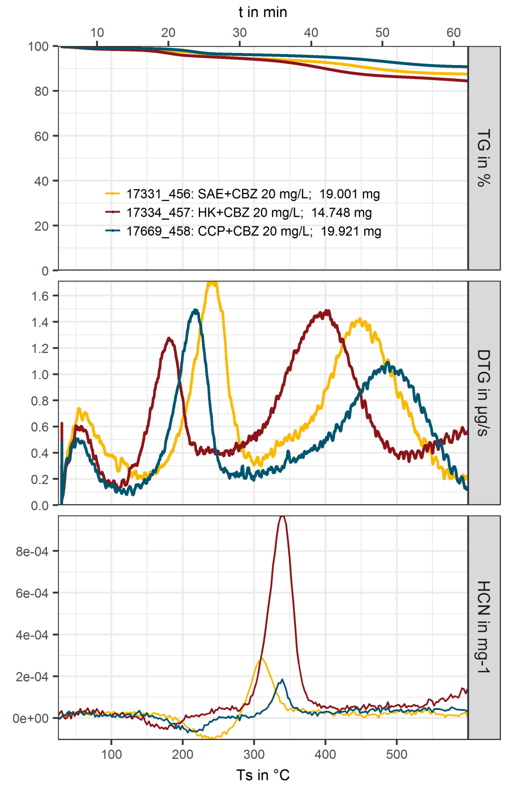

Figure S4. TGA-FTIR results for the highest loadings of carbamazepine on the three activated carbons. The figure corresponds to Figure 2(a). Release rates of hydrogen cyanide are shown normalised to sample mass.

3.2 Release graphs for all decomposition products of the 2nd mass loss stage

This section covers all decomposition products in the 2nd mass loss stage observed by TED-GC/MS. Data represent double determinations, comparing the three activated carbon systems with 4.5 % carbamazepine and their release from the unloaded adsorbents. Furthermore, the release of the proposed substances from the SAE Super systems depending on the carbamazepine loading, represented by the 2nd stage's mass loss, is provided. Values in the following figures (S5 ... S58) are normalised to sample mass and error bars represent the range of the duplicate determination. Numbering of the substances is based on retention time and can be found the spreadsheet *Structures_by_TED-GC-MS*.

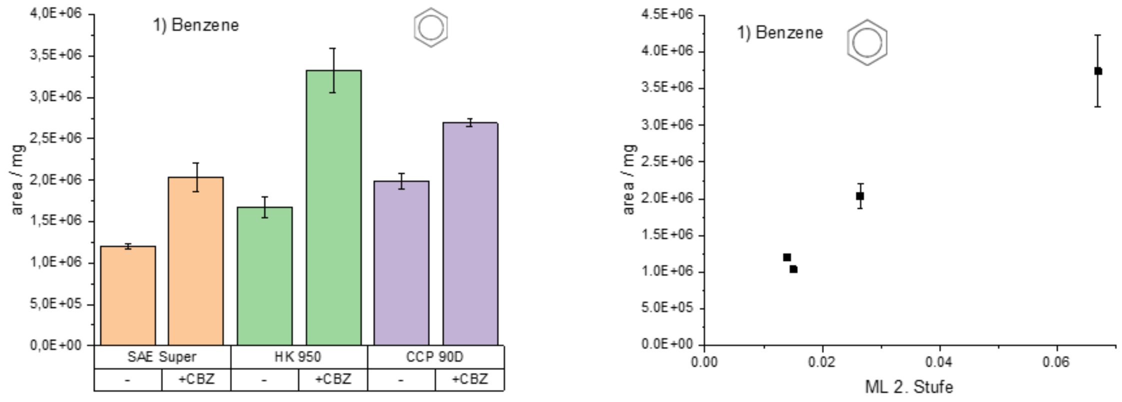

Figure S5. Benzene (1)

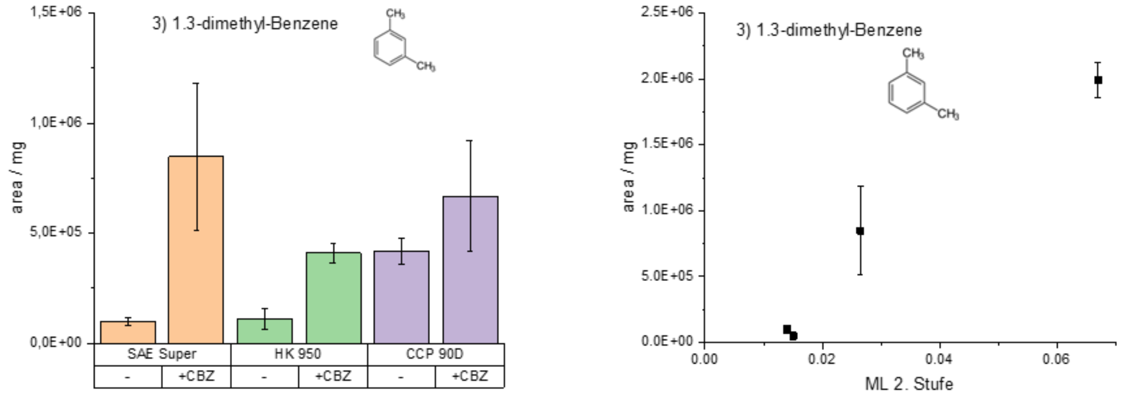

Figure S6. 1,3-dimethyl-Benzene (3)

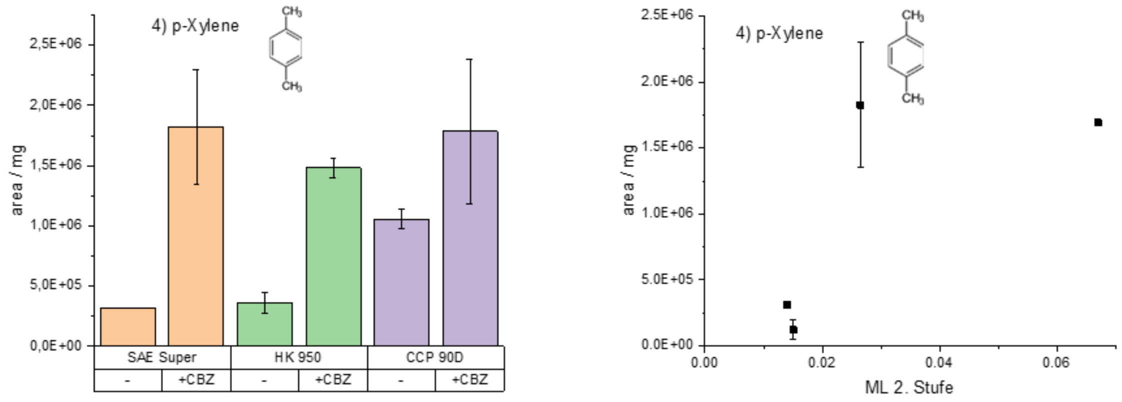

Figure S7. p-Xylene (4)

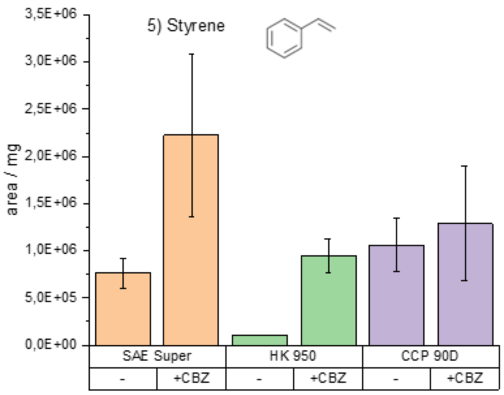

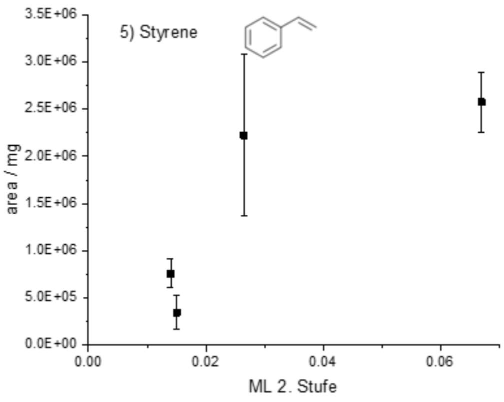

Figure S8. Styrene (5)

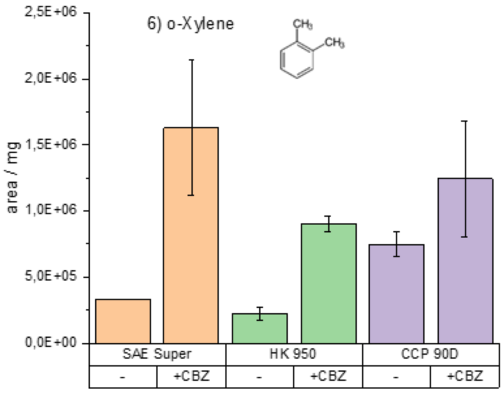

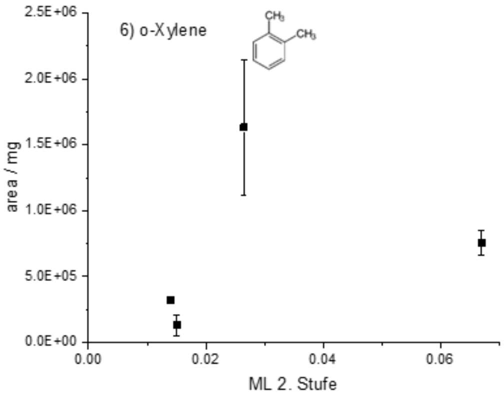

Figure S9. o-Xylene (6)

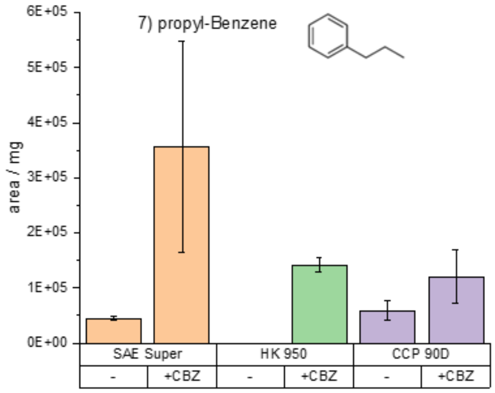

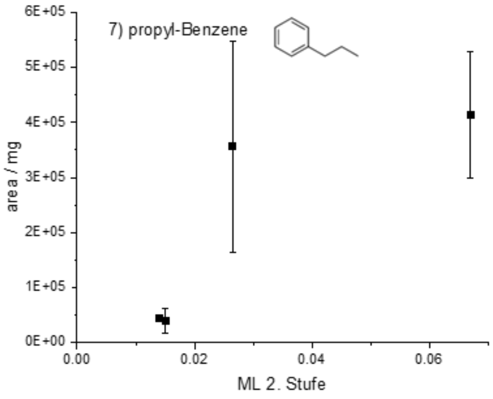

Figure S10. Propyl-Benzene (7)

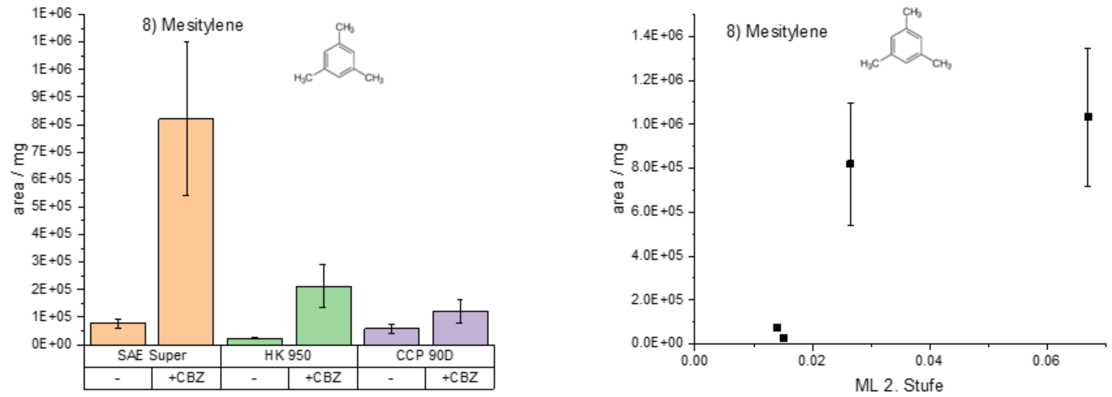

Figure S11. Mesitylene (8)

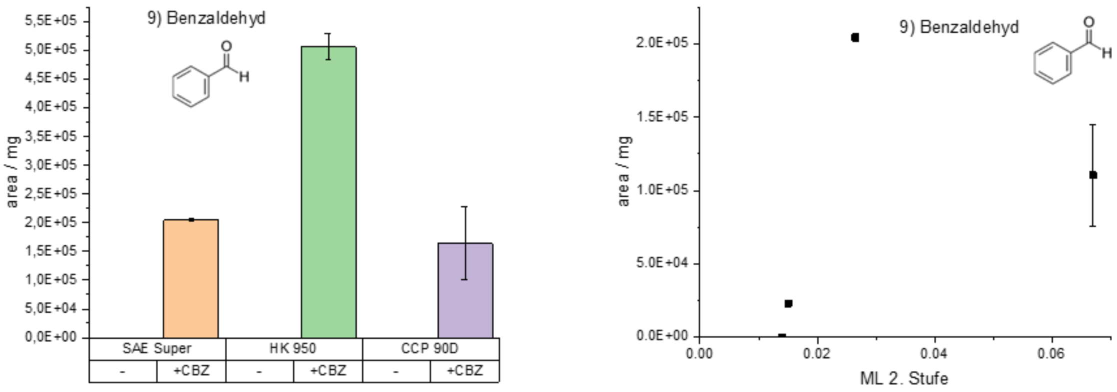

Figure S12. Mesitylene (9)

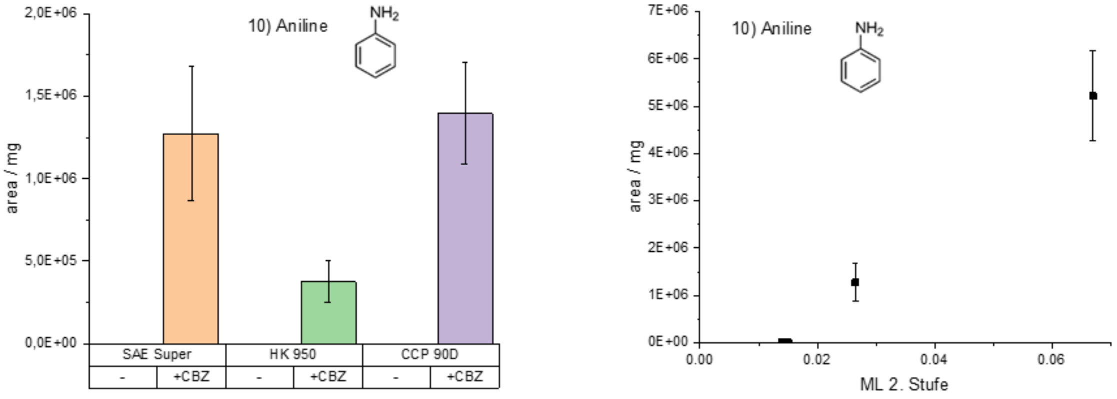

Figure S13. Mesitylene (10)

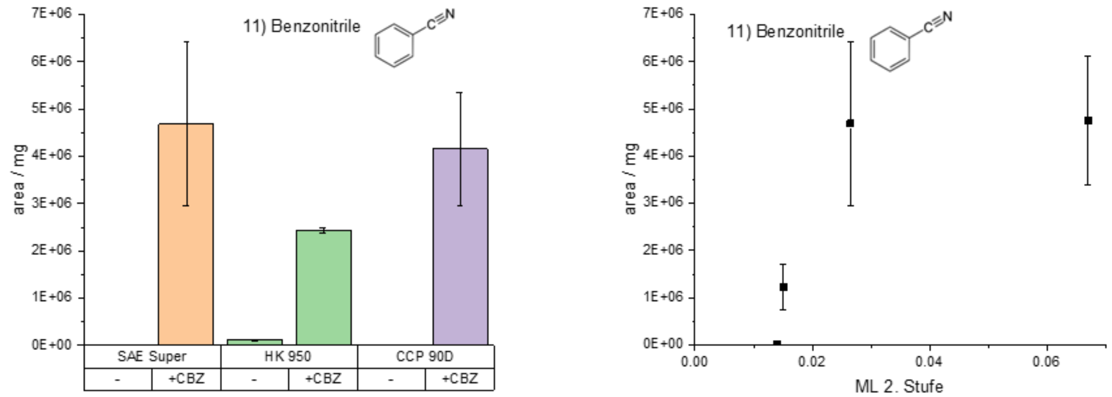

Figure S14. Benzonitrile (11)

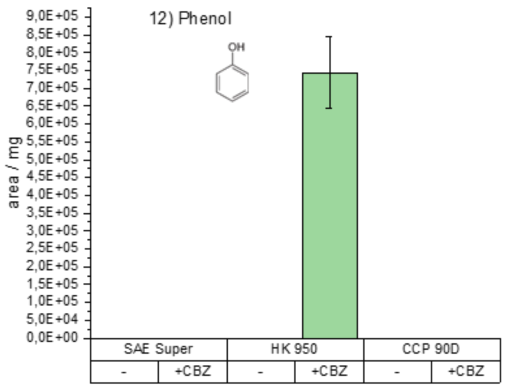

Figure S15. Phenol (12)

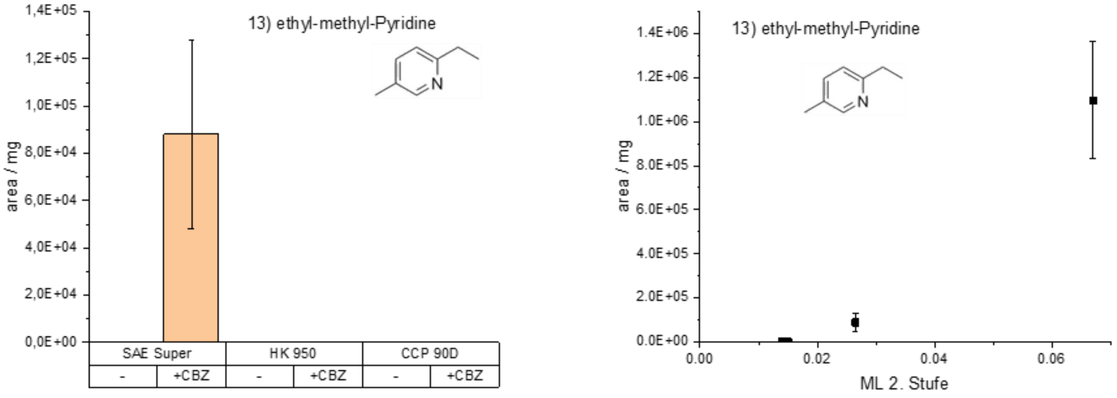

Figure S16. ethyl-methyl-Pyridine or m-Ethylanaline (13)

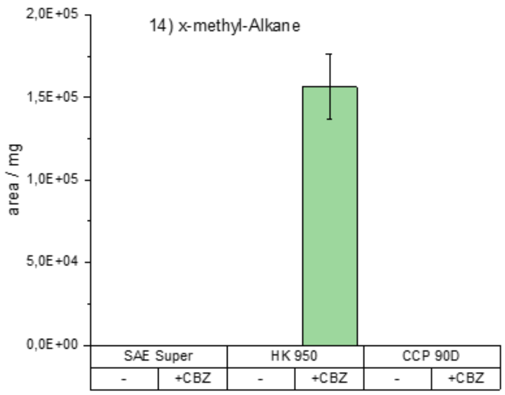

Figure S17. x-methyl-Alkane (14)

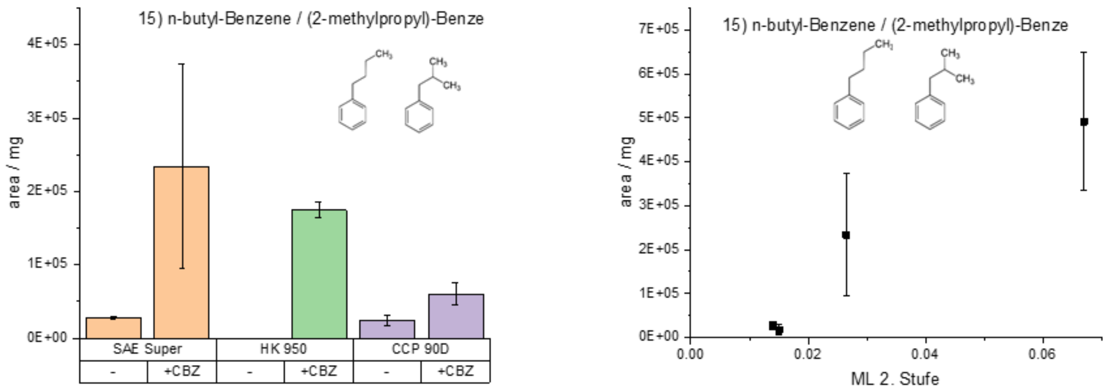

Figure S18. n-butyl-Benzene or (2-methylpropyl)-Benzene (15)

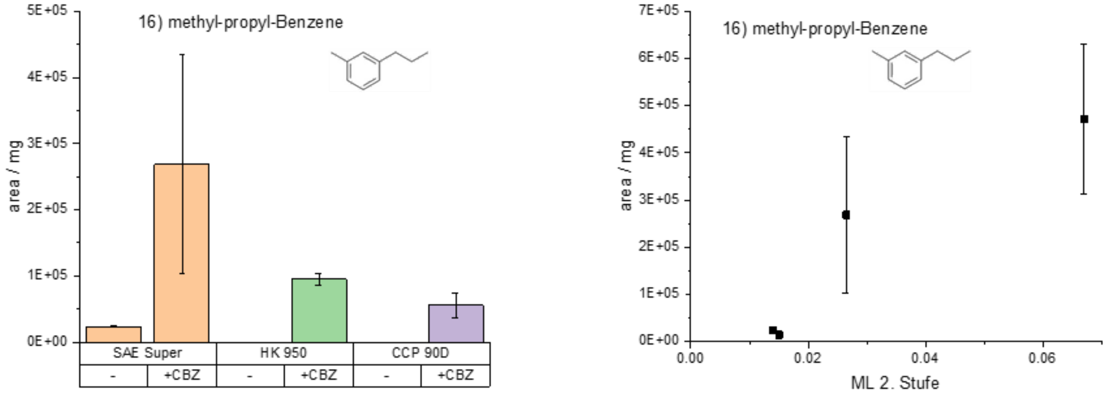

Figure S19. methyl-propyl-Benzene (16)

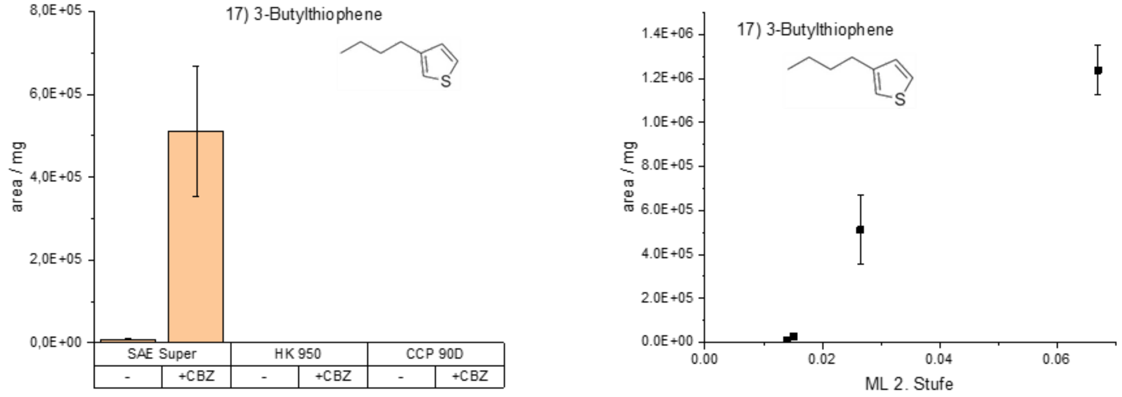

Figure S20. 3-Butylthiophene (17)

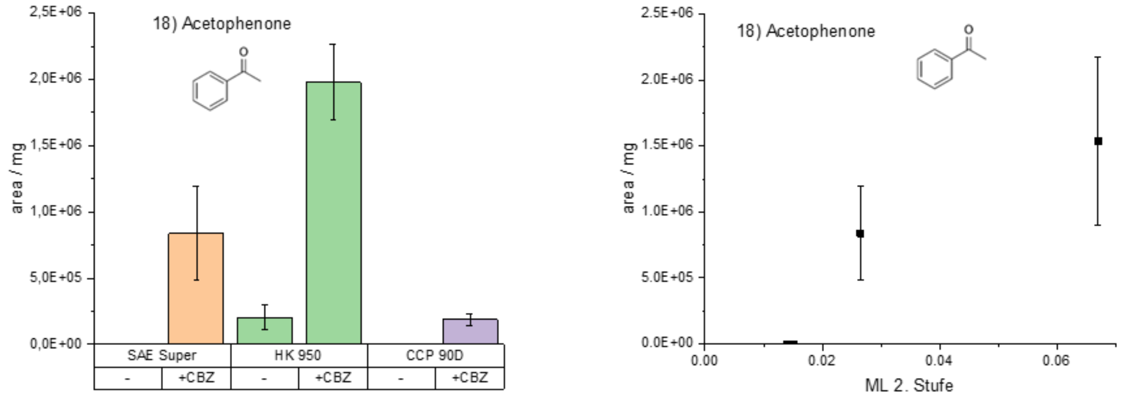

Figure S21. Acetophenone (18)

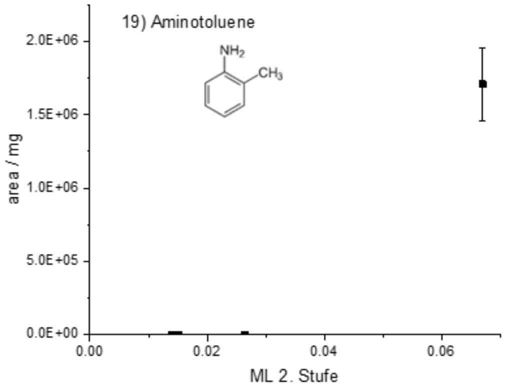

Figure S22. Acetophenone (19)

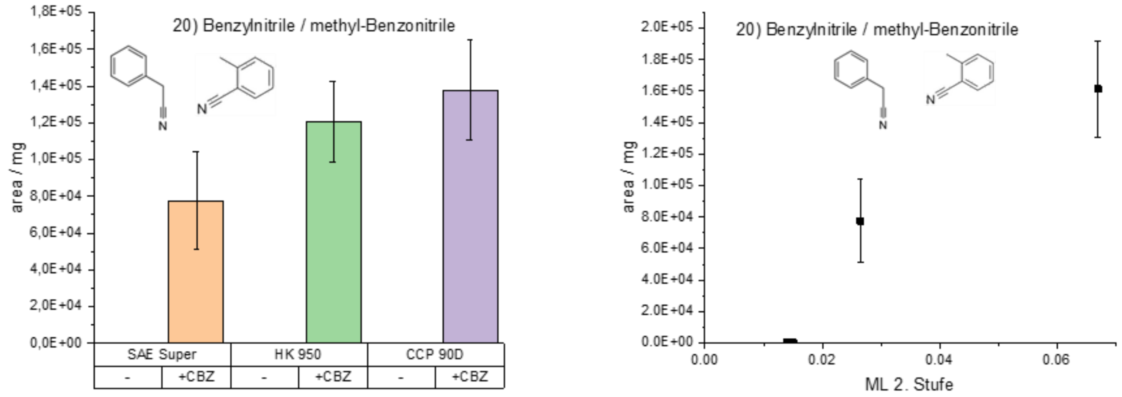

Figure S23. Benzylnitrile or methyl-Benzonitril (20)

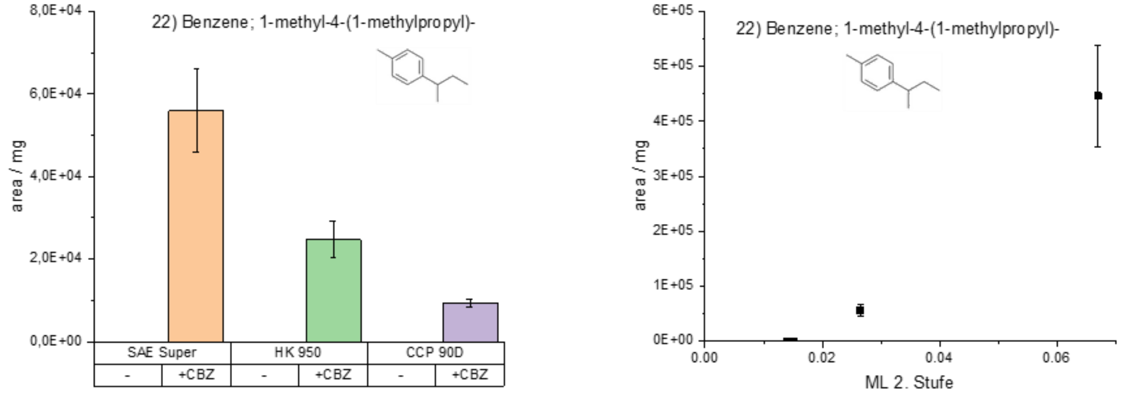

Figure S24. Benzene, 1-methyl-4-(1-methylpropyl)- (22)

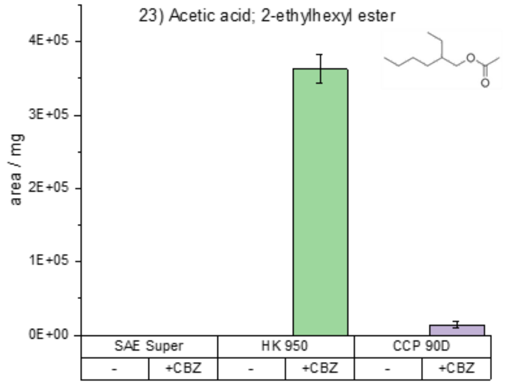

Figure S25. Acetic acid, 2-ethylhexyl ester (23)

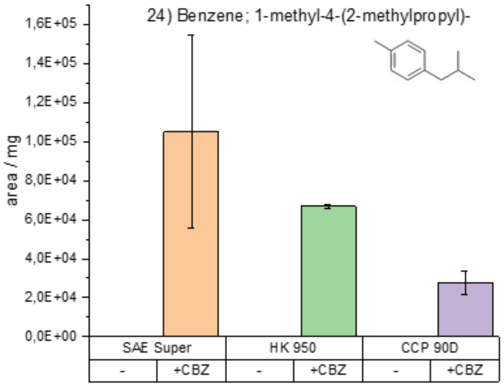

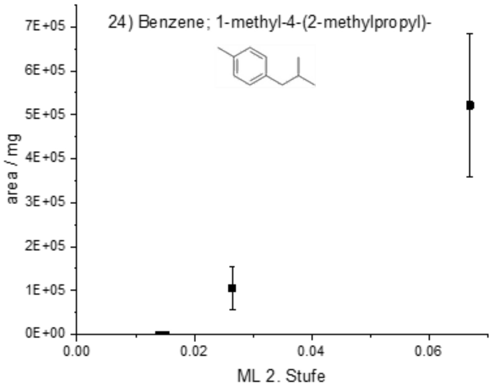

Figure S26. Benzene, 1-methyl-4-(2-methylpropyl)- (24)

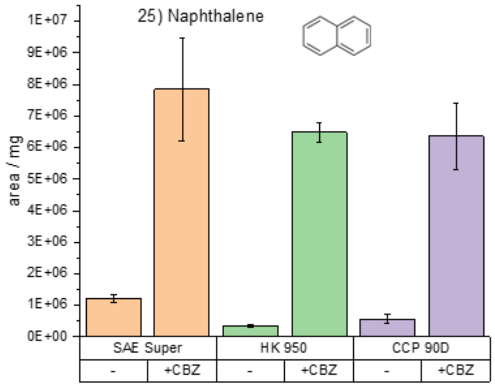

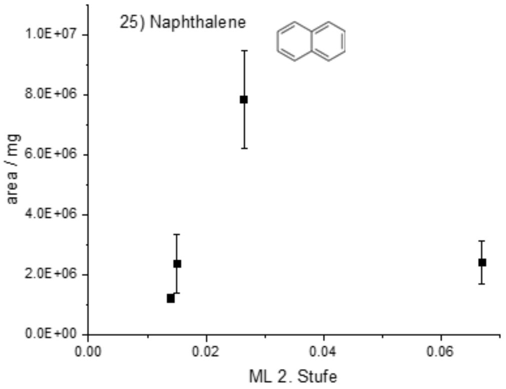

Figure S27. Naphthalene (25)

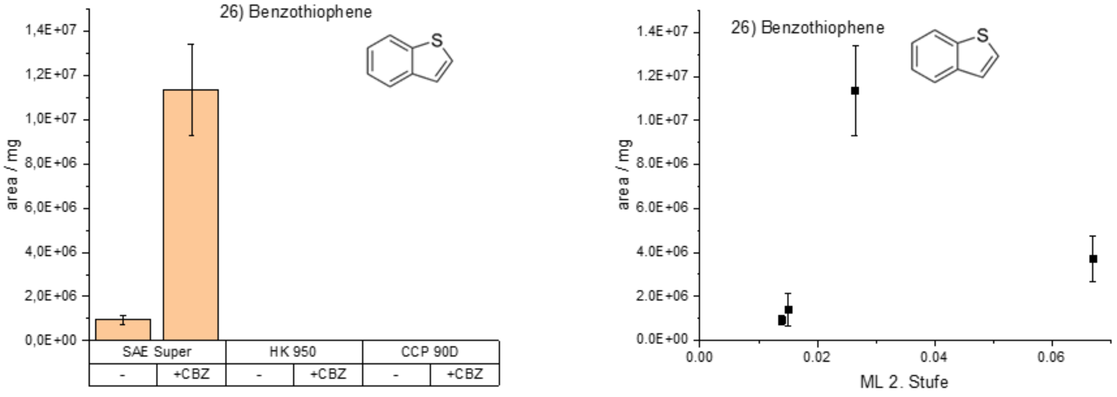

Figure S28. Benzothiophene (26)

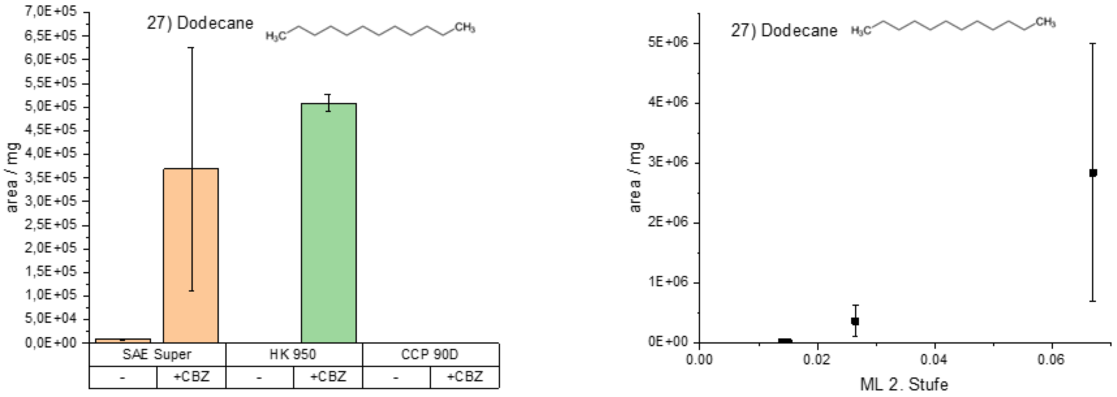

Figure S29. Dodecane (27)

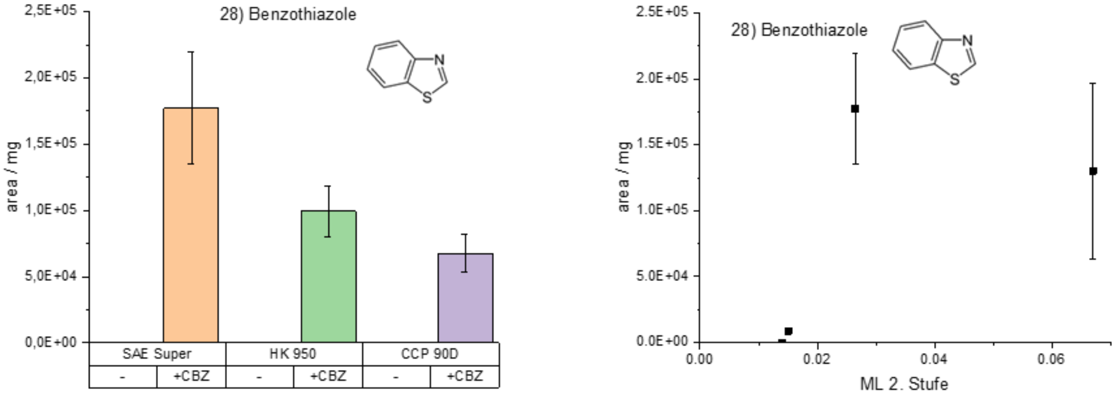

Figure S30. Benzothiazole (28)

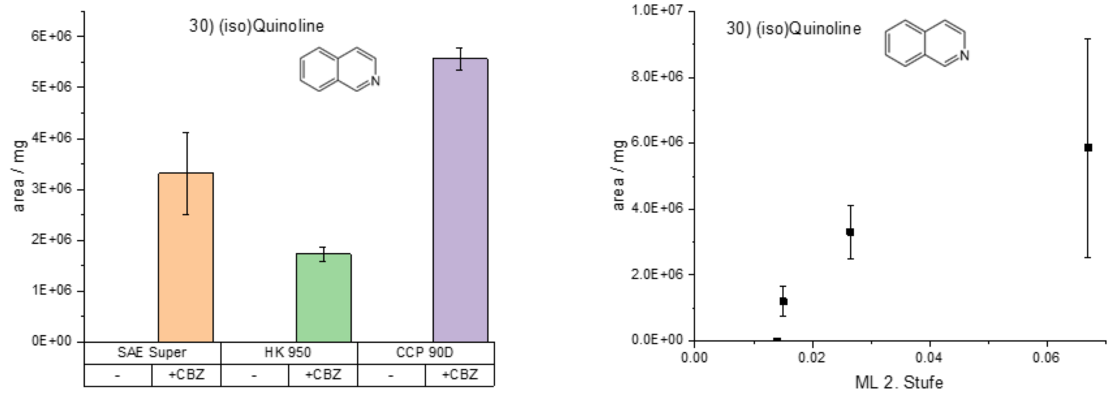

Figure S31. (iso)Quinoline (30)

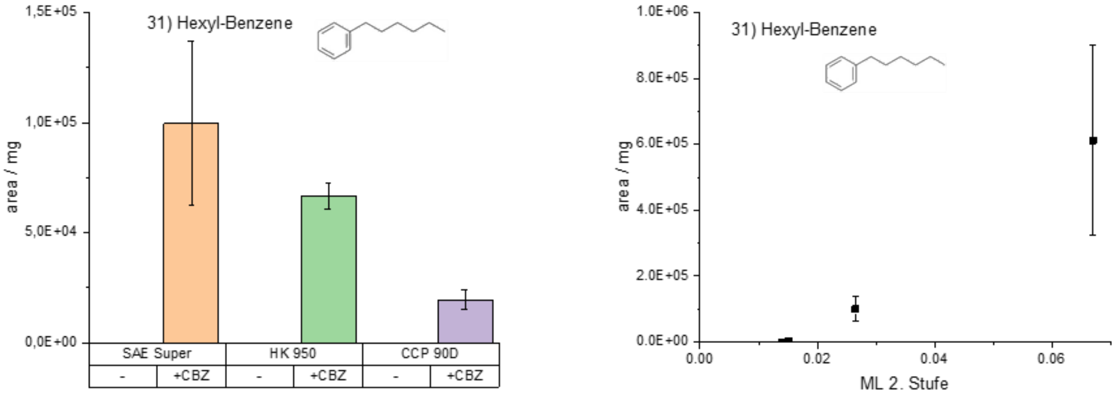

Figure S32. Hexyl-Benzene (31)

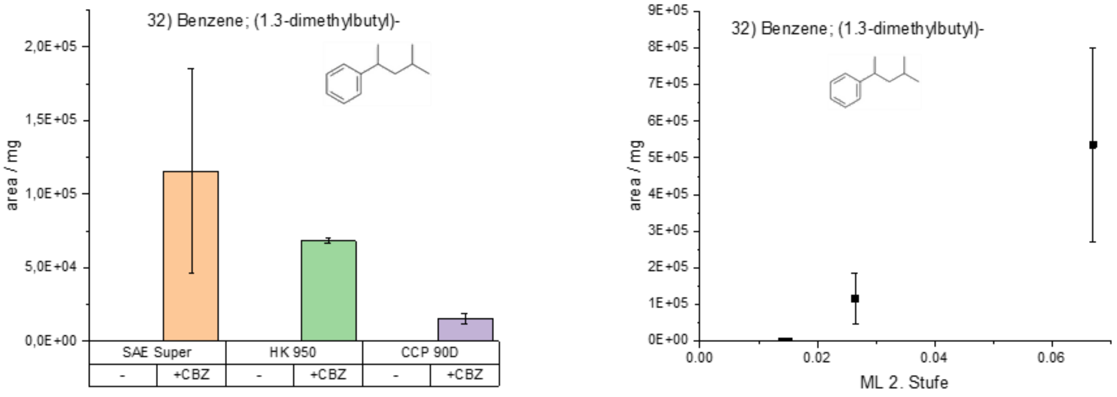

Figure S33. Benzene, (1,3-dimethylbutyl)- (32)

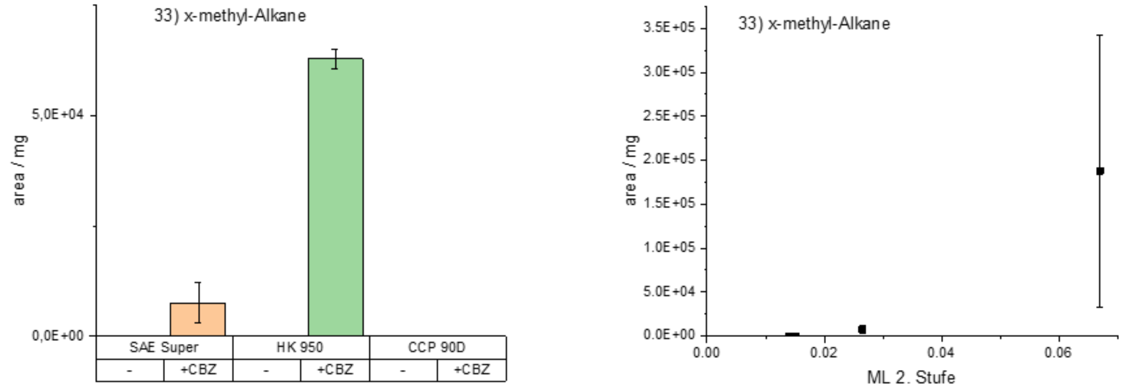

Figure S34. x-methyl-Alkane (33)

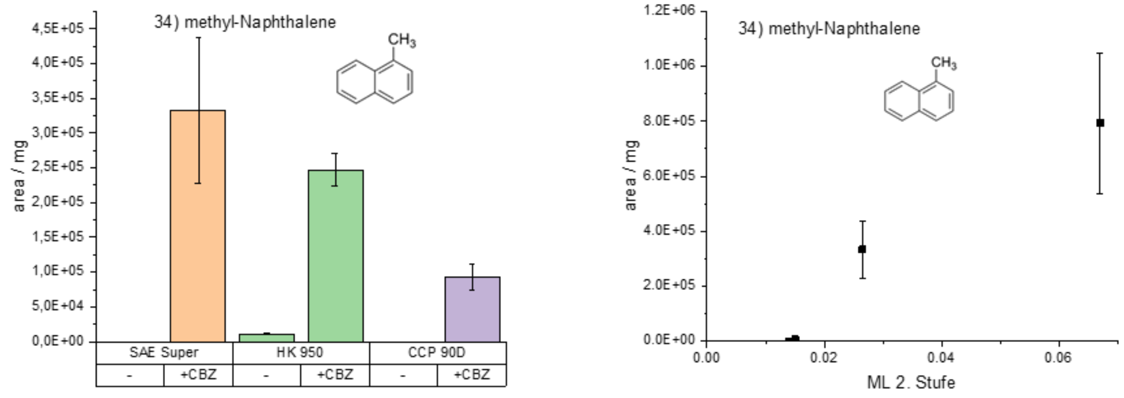

Figure S35. methyl-Naphthalene (34)

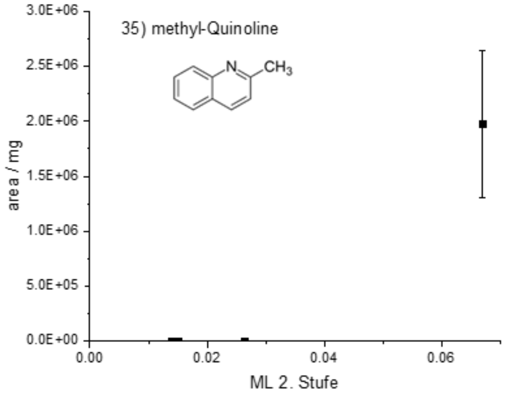

Figure S36. methyl-Quinoline (35)

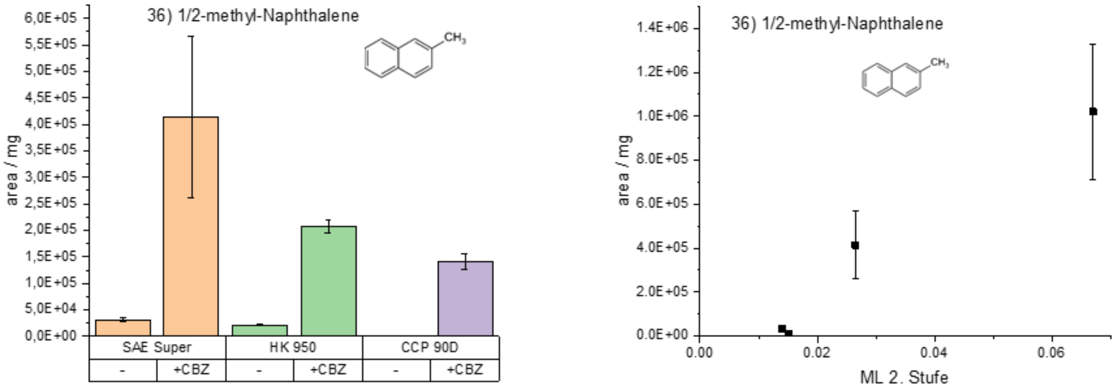

Figure S37. 1- or 2-methyl-Naphthalene (36)

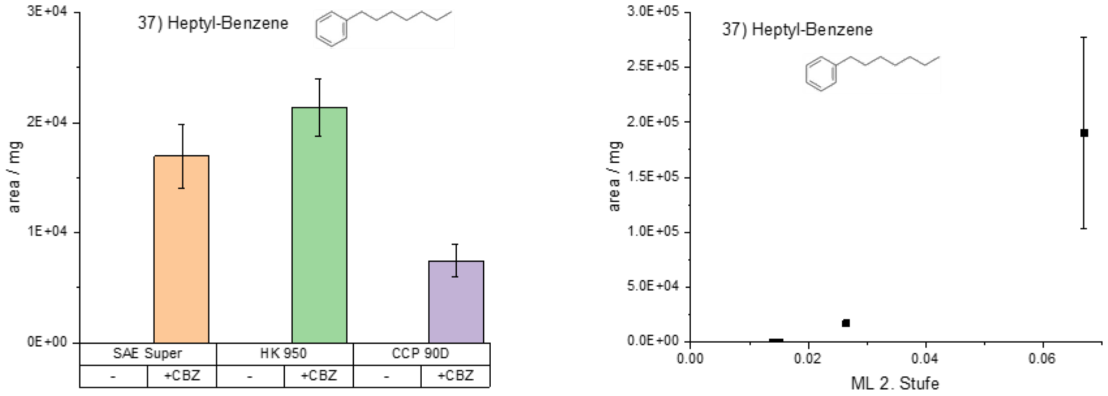

Figure S38. Heptyl-Benzene (37)

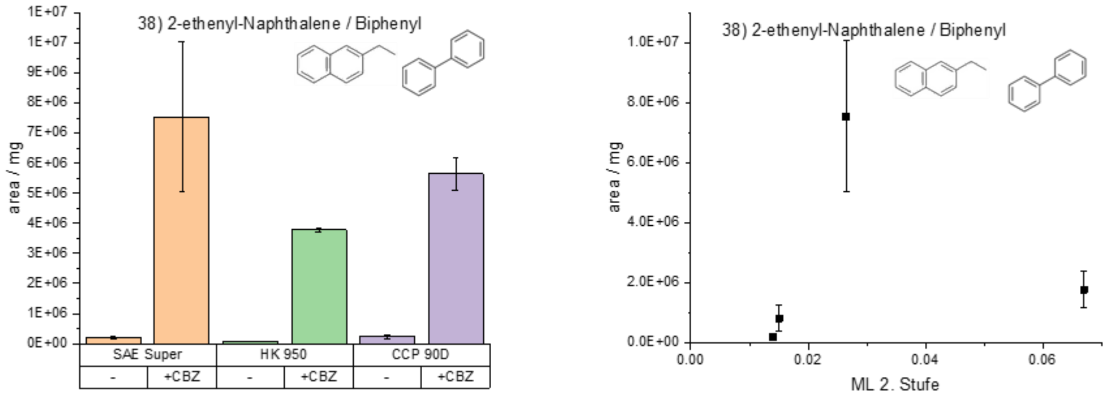

Figure S39. 2-ethenyl-Naphthalene or Biphenyl (38)

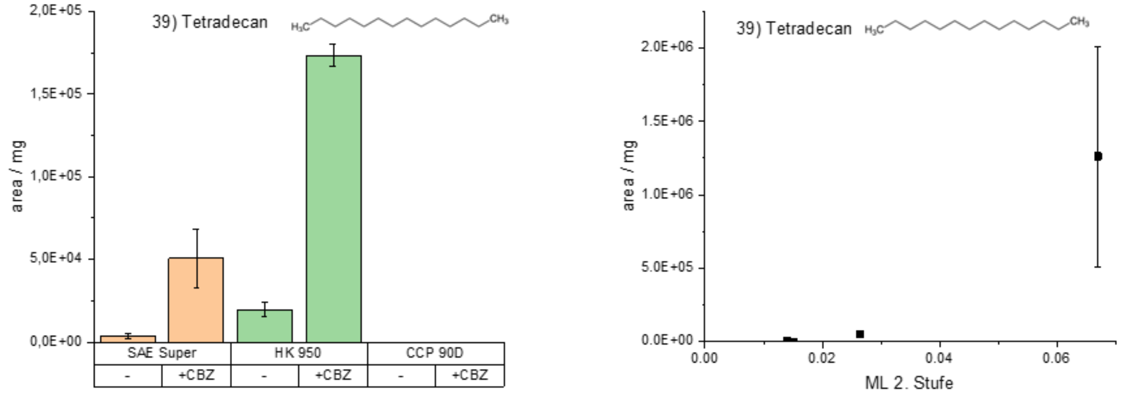

Figure S40. Tetradecane (39)

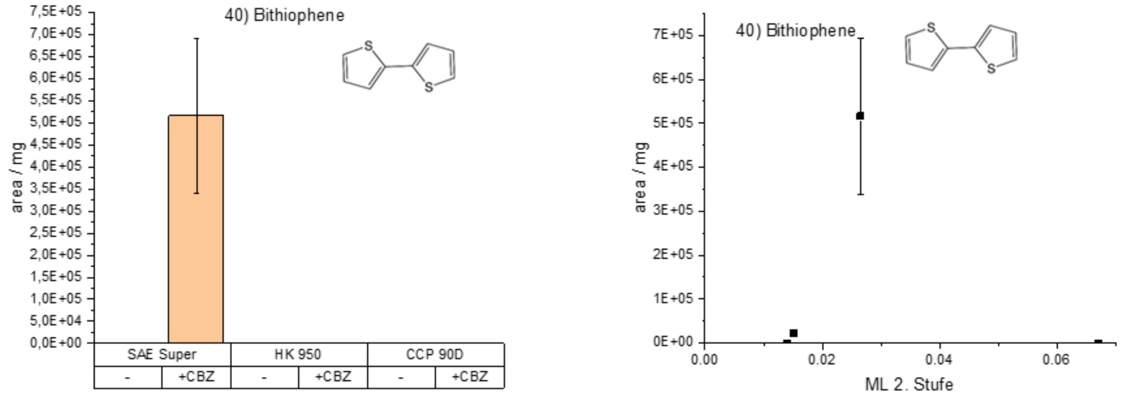

Figure S41. Bithiophene (40)

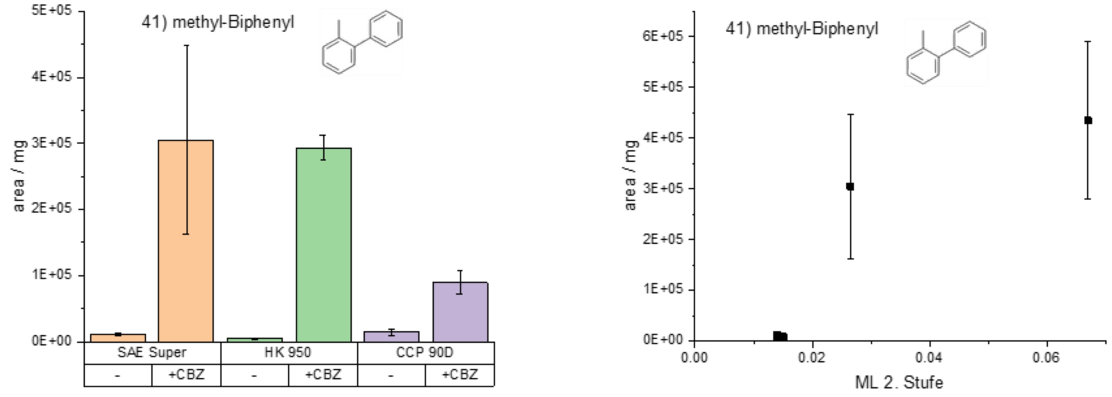

Figure S42. methyl-Biphenyl (41)

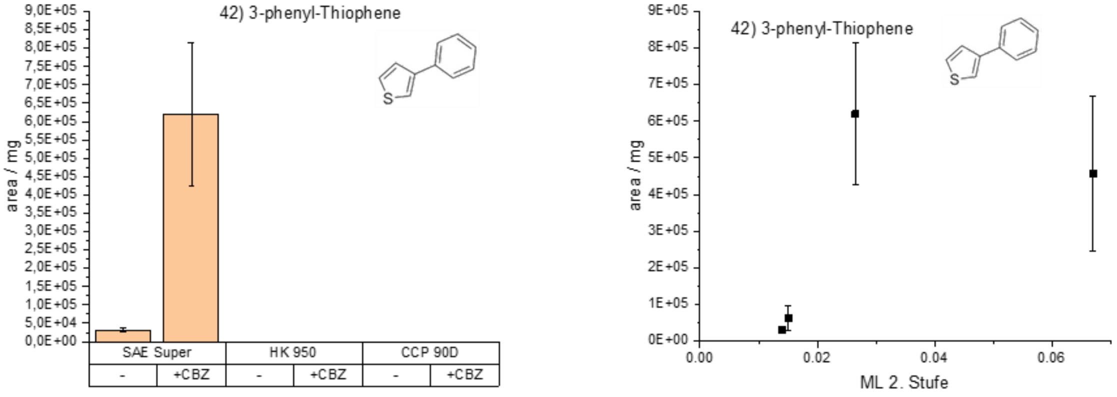

Figure S43. 3-phenyl-Thiophene (42)

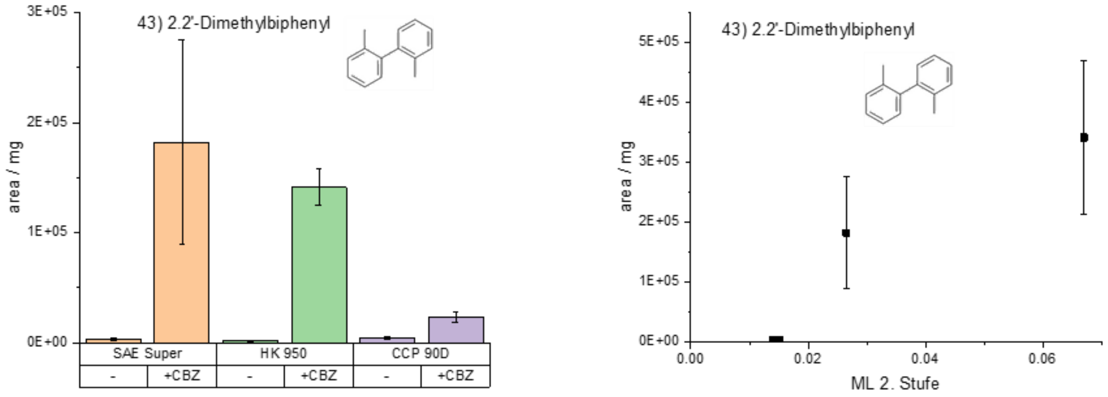

Figure S44. 2,2'-Dimethylbiphenyl (43)

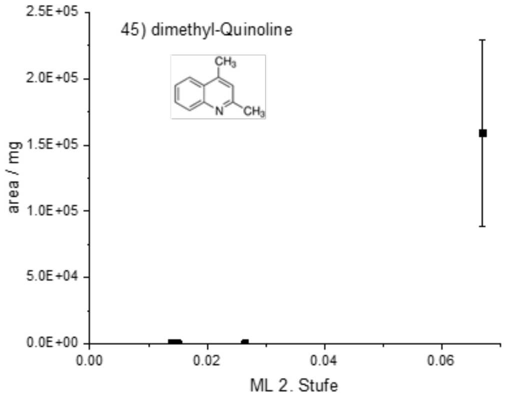

Figure S45. dimethyl-Quinoline (45)

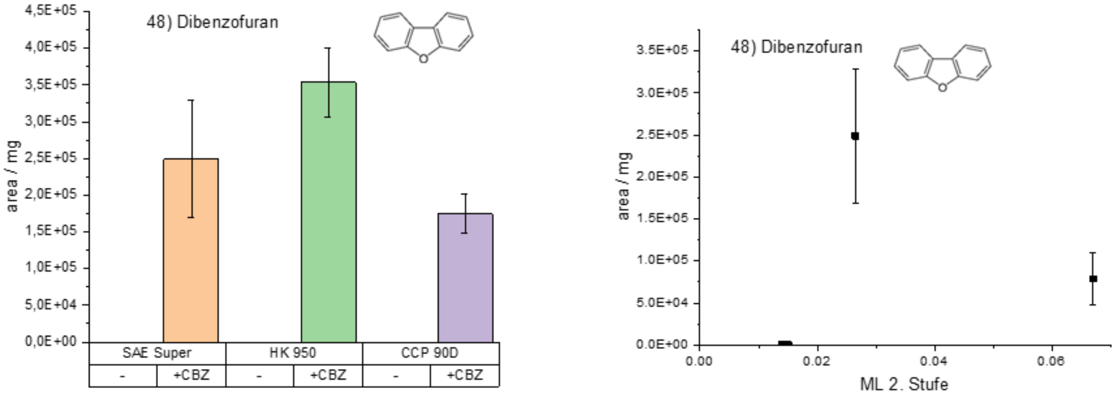

Figure S46. Dibenzofuran (48)

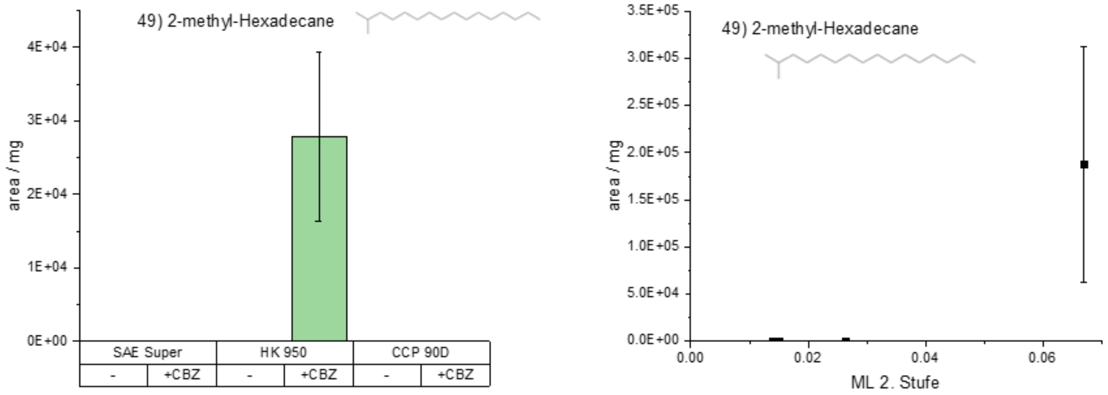

Figure S47. 2-methyl-Hexadecane (49)

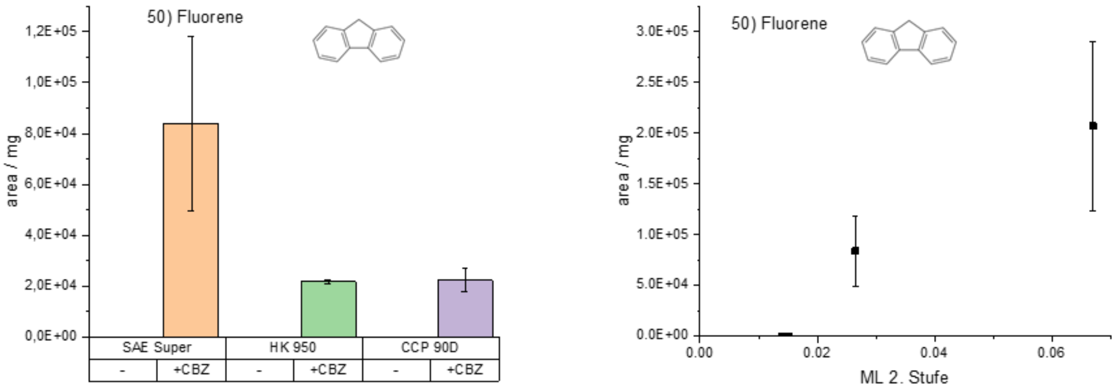

Figure S48. Fluorene (50)

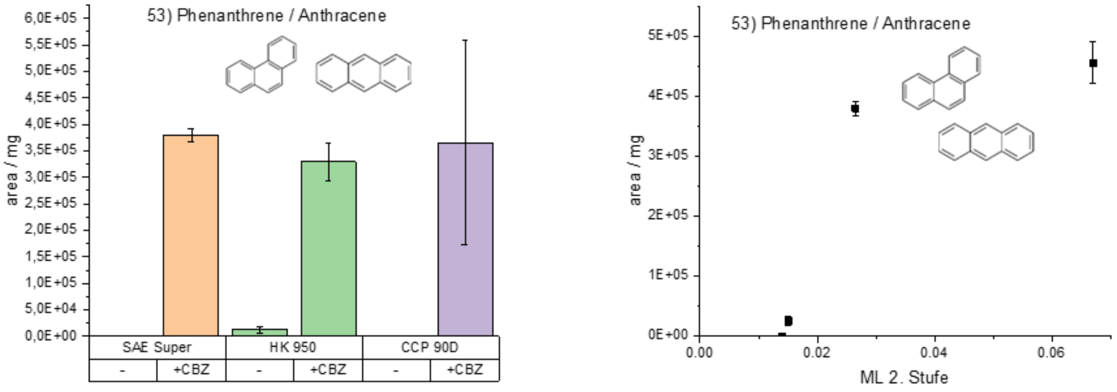

Figure S49. Phenanthrene or Anthracene (53)

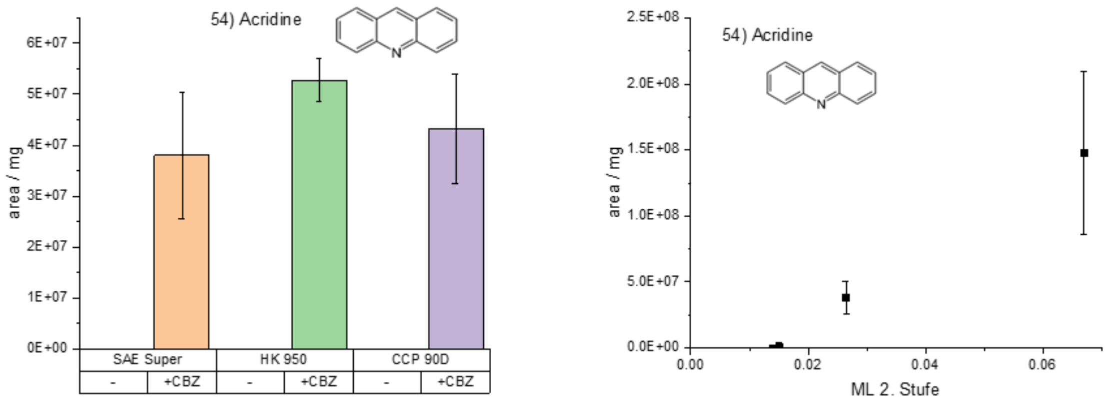

Figure S50. Acridine (54)

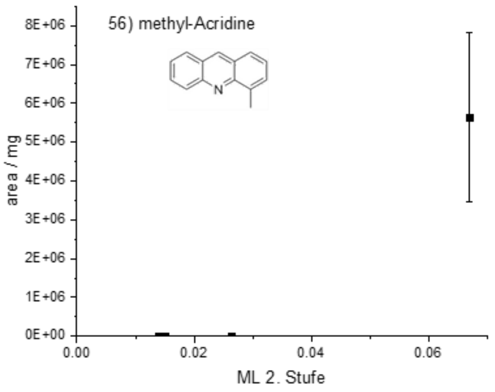

Figure S51. (4-)methyl-Acridine (56)

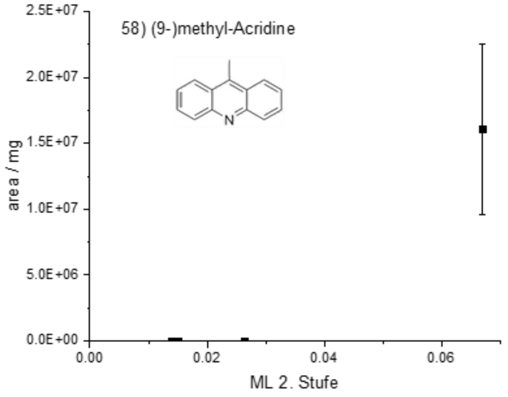

Figure S52. (9-)methyl-Acridine (58)

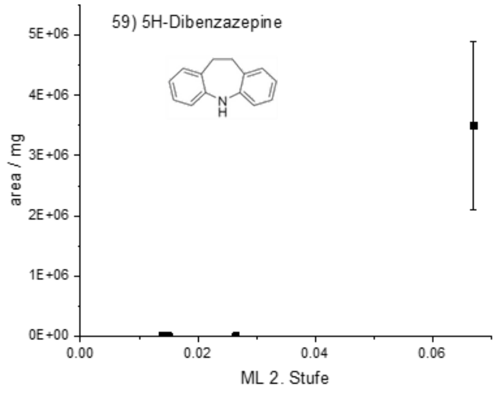

Figure S53. 5H-Dibenzazepine, 10,11-dihydro- (59)

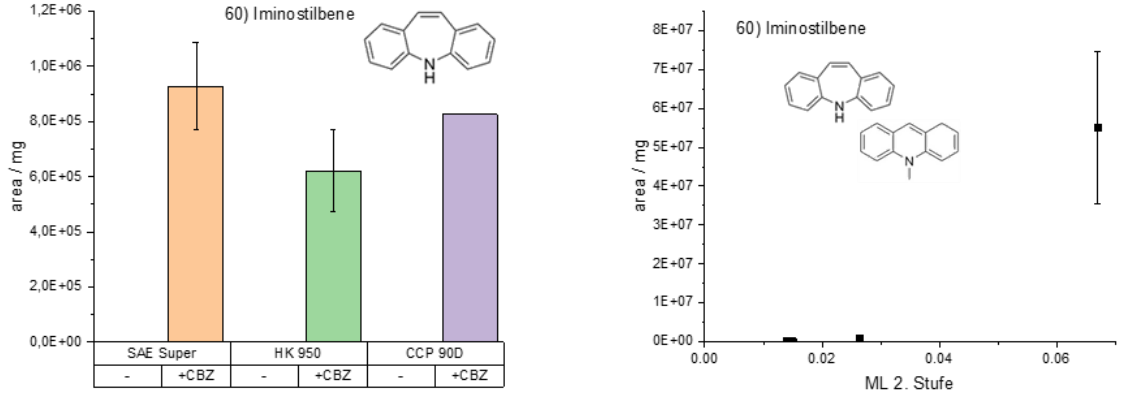

Figure S54. Dibenzazepine, "Iminostilbene" (60)

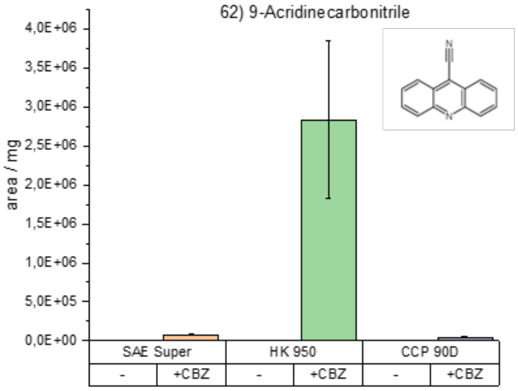

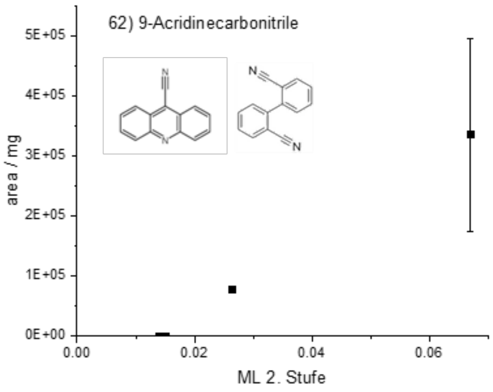

Figure S55. 9-Acridinecarbonitrile or Biphenyldicarbonitrile (62)

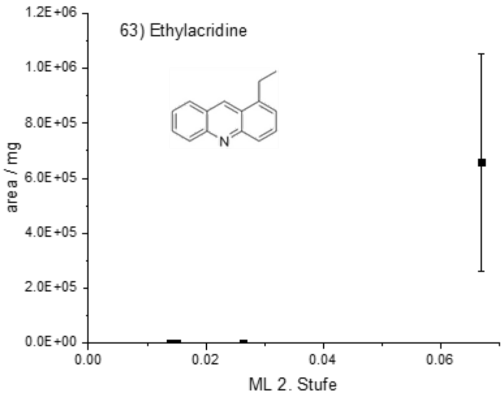

Figure S56. Ethylacridine (63)

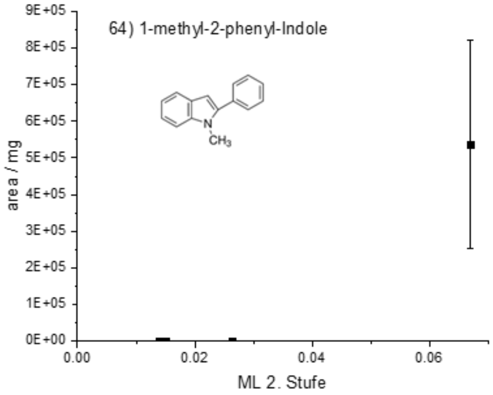

Figure S57. 1-methyl-2-phenyl-Indole (64)

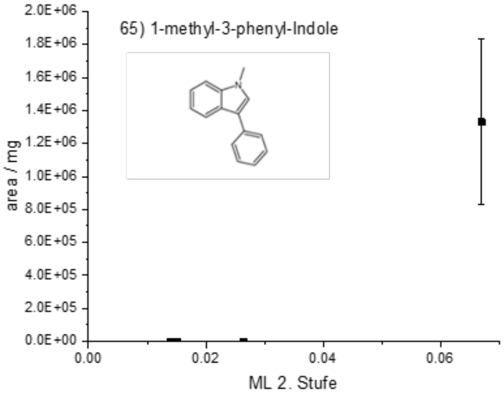

Figure S58. 1-methyl-3-phenyl-Indole (65)

4 SEM comparisons

Scanning electron microscopy (SEM) images were recorded of the HK 950 and the CCP 90D systems exemplary for loaded and unloaded samples (Figures S59&S60). The samples were treated identically, but with or without carbamazepine in solution (see Materials and Methods). The images do not show differences in the activated carbon particle's morphology. This confirms that adsorption takes place in the pores, on the inner surface of the particles, even for large amounts of adsorbate.

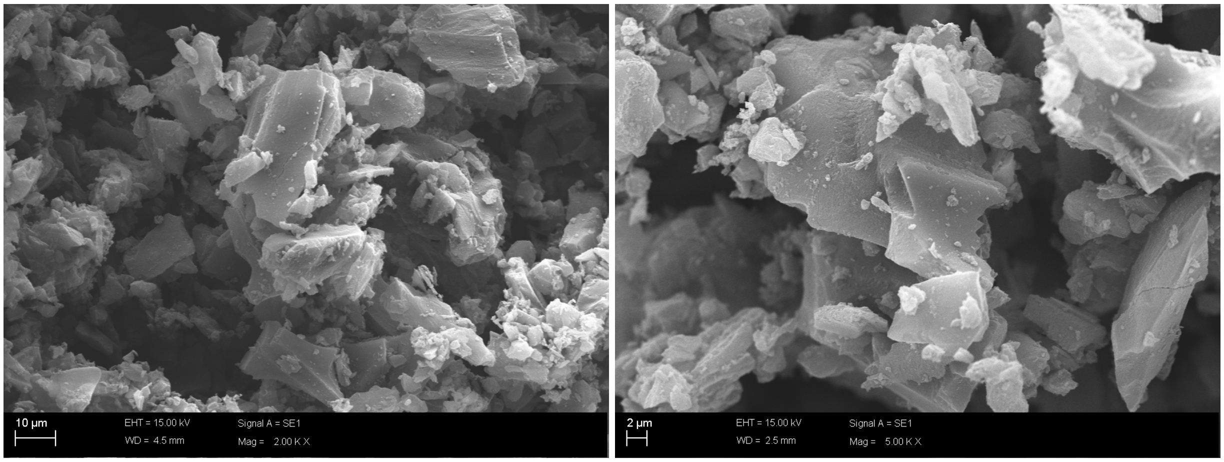

Figure S59. SEM images of HK 950 systems. Left hand side: HK and right hand side: HK+CBZ-15.

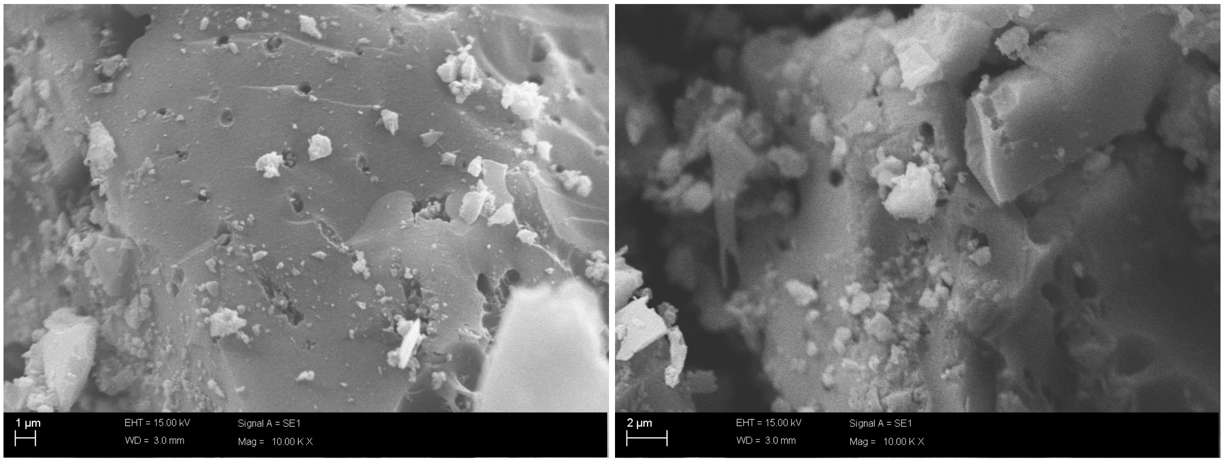

Figure S60. SEM images of CCP 90D systems. Left hand side: CCP and right hand side: CCP+CBZ-12.

5 DRIFTS comparisons

Diffuse reflectance infrared fourier transform spectroscopy (DRIFTS) have been applied to all three activated carbons unloaded and loaded with carbamazepine. No differences in the spectra could be observed for the Systems with SAE Super and CCP 90D (Figure S61). The negative bands are spectroscopic effects due to the strong absorption of the activated carbons (about 4 % in KBr). Figure S62a shows the spectra for the HK 950 systems. These spectra are typical for chemical activated, lignocellulose based materials.[4] Furthermore, additional bands occure in the systems loaded with carbamazepine. Figure S62b highlights the wavelength region between 1800 and 650 cm^{-1} with the subtraction sepctrum of loaded and unloaded HK 950 as well as the spectrum of pure carbamazepine. Distinct bands can be found and attributed to intact carbamazepine molecules on the surface of the activated carbon. However, it is related to the spectral properties of HK 950 that carbamazepine is detectable by DRIFTS.

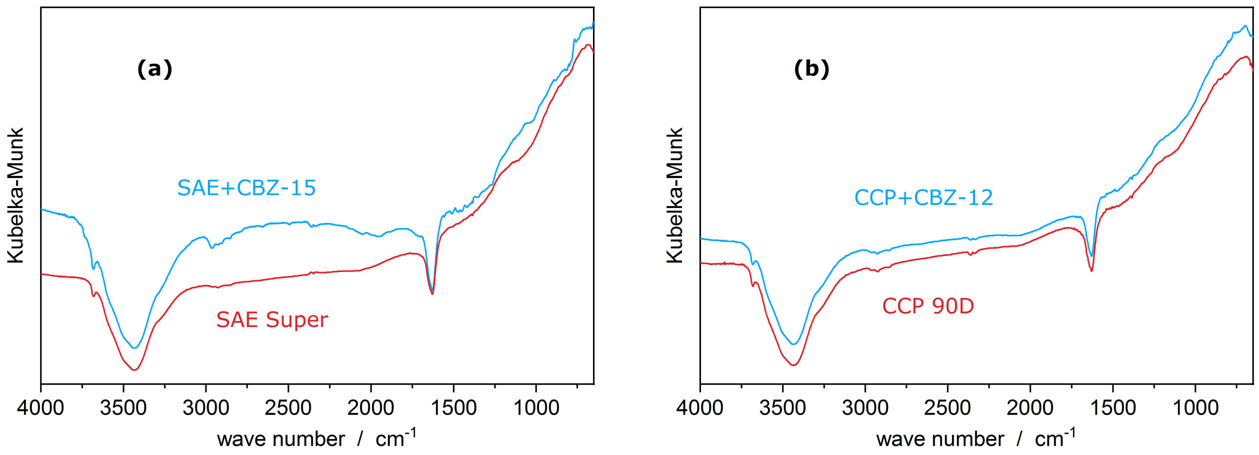

Figure S61. DRIFT spectra of (a) loaded and unloaded SAE Super, (b) loaded and unloaded CCP 90D.

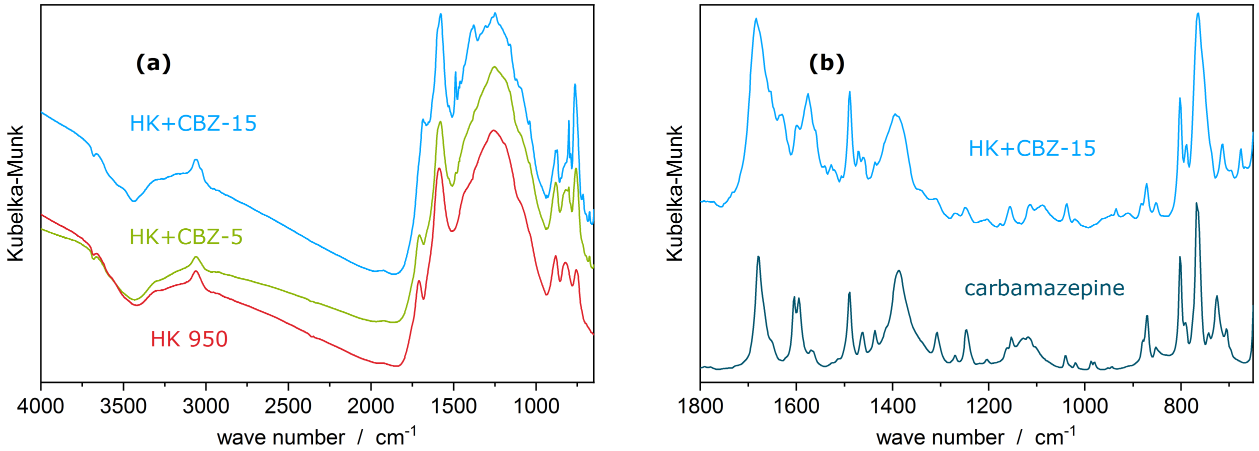

Figure S62. DRIFT spectra of (a) loaded and unloaded HK 950 systems, (b) subtraction spectrum of HK+CBZ-15 and unloaded HK 950 is compared with the spectrum of pure carbamazepine in a qualified wave length region.

References

1. Dittmann, D. Experimental raw data for "Specific adsorption sites and conditions derived by thermal decomposition of activated carbons and adsorbed carbamazepine", DOI: https://doi.org/10.5281/zenodo.3716316 (2020).
2. Pinto, M. A. L. *et al.* Thermoanalytical studies of carbamazepine: Hydration/dehydration, thermal decomposition, and solid phase transitions. *Braz. J. Pharm. Sci.* **50**, 877–884, DOI: https://doi.org/10.1590/S1984-82502014000400023 (2014).
3. Fischer, G., Geith, J., Klapötke, T. M. & Krumm, B. Synthesis, Properties and Dimerization Study of Isocyanic Acid. *Zeitschrift für Naturforschung B* **57**, 19–24, DOI: https://doi.org/10.1515/znb-2002-0103 (2002).
4. Puziy, A. M., Poddubnaya, O. I., Martínez-Alonso, A., Suárez-García, F. & Tascón, J. M. D. Surface chemistry of phosphorus-containing carbons of lignocellulosic origin. *Carbon* **43**, 2857–2868, DOI: https://10.1016/j.carbon.2005.06.014 (2005).

Acknowledgements

We thankfully acknowledge Frederick Zietzschmann for providing the activated carbons and the information about their characterisation, as well as Ute Kalbe, Jutta Jakobs and Christiane Weimann for very kindly XRF, LC-MS/MS and SEM measurements, respectively. We thank Glen J Smales for the very helpful proofreading. Last but not least we acknowledge support by the German Research Foundation and the Open Access Publication Fund of TU Berlin.
